# Supplementary material for: Chronic hepatitis B virus case-finding in UK populations born abroad in intermediate or high endemicity countries: an economic evaluation
Source: BMJ Open. 2019 Jun 28;9(6):e030183. doi: 10.1136/bmjopen-2019-030183 (PMC6609059; doi:10.1136/bmjopen-2019-030183)
Supplement: Supplementary data [file bmjopen-2019-030183supp001.pdf]

Supplementary Materials

Supplementary Table 1: Annual transition probability matrix for people who enter the model as HBsAg+ and HBeAg+ derived from Shepherd(1) and Marcellin(2)

**To:**<br>**From:**	**HBsAg seroconverted**	**HBeAg seroconverted**	**CHB HBeAg+ active disease**	**CC**	**DC**	**HCC**	**LT1**	**LT2**	**Dead[+]**
**HBsAg seroconverted**	#	0	0	0	0	0.00005	0	0	0
**HBeAg seroconverted**	0.02	#	0.03	0.01	0	0.005	0	0	0
**CHB HBeAg+ active disease no treatment**	0.0175	0.05	#	0.05	0	0.005	0	0	0.0035
**CHB HBeAg+ active disease or CC on treatment**									
**Treatment response with peglyated interferon**	0.0175	0.20	#	0.05	0	0.005	0	0	0.0035
**Treatment response with tenofovir**	0.0175	0.054	#	0.05	0	0.005	0	0	0.0035
**Compensated cirrhosis (CC) no treatment**	0	0.05	0	#	0.05	0.025	0	0	0.051
**Decompensated cirrhosis (DC)**	0	0	0	0	#	0.025	0.03	0	0.39
**Hepatocellular carcinoma (HCC)**	0	0	0	0	0	#	0	0	0.56
**Liver transplant – first year (LT1)**	0	0	0	0	0	0	#	0	0.21
**Liver transplant – subsequent years (LT2)**	0	0	0	0	0	0	0	#	0.057

[+]an age-adjusted general population mortality is added to this amount; #, indicates the residual row probability; all values are converted to a Dirichlet distribution by assuming an effective sample size of 200 for each row(3)

Supplementary Table 2: Annual transition probability matrix for people who enter the model as HBsAg+ and HBeAg- derived from Shepherd(1) and Marcellin(2)

To: From:	HBsAg seroconverted	HBeAg seroconverted	CHB HBeAg- active disease	CC	DC	HCC	LT1	LT2	Dead+
**HBsAg seroconverted**	#	0	0	0	0	0.00005	0	0	0
**HBeAg seroconverted**	0.0175	#	0.03	0.01	0	0.005	0	0	0
**CHB HBeA- active disease no treatment**	0	0.015	#	0.05	0	0.005	0	0	0.0035
**CHB HBeAg- active disease or CC on treatment**									
**Treatment response with peglyated interferon**	0	0.75	#	0.05	0	0.005	0	0	0.0035
**Treatment response with tenofovir**	0	0.023	#	0.05	0	0.005	0	0	0.0035
**Decompensated cirrhosis (DC)**	0	0	0	0	#	0.025	0.03	0	0.39
**Hepatocellular carcinoma (HCC)**	0	0	0	0	0	#	0	0	0.56
**Liver transplant – first year (LT1)**	0	0	0	0	0	0	#	0	0.21
**Liver transplant – subsequent years (LT2)**	0	0	0	0	0	0	0	#	0.057

+an age-adjusted general population mortality is added to this amount; #, indicates the residual row probability; all values are converted to a Dirichlet distribution by assuming an effective sample size of 200 for each row(3)

Supplementary Table 3: Utility values from Shepherd(1) and Takeda(4)

Utility	Mean decrement	95% interval of sampled range^
**Age**		-
**0-44**	0.09	-
**45-54**	0.15	-
**55-64**	0.20	-
**65-74**	0.22	-
**75+**	0.27	-
**HBsAg-**	0	-
**HBeAg+ seroconverted / HBeAg- ALT/DNA low**	0	-
**HBeAg+ / HBeAg- active disease**	0.04	0.023-0.062
**Compensated cirrhosis**	0.44	0.34-0.55
**Decompensated cirrhosis**	0.54	0.43-0.73
**Hepatocellular carcinoma**	0.54	0.43-0.73
**Liver transplant (first year)**	0.54	0.43-0.73
**Liver transplant (subsequent years)**	0.32	0.22-0.43

Utility values are calculated by subtracting appropriate decrements from 1; ^Sampled values from the probabilistic sensitivity analysis using a beta distribution

Supplementary Figure 1. Cost-effectiveness acceptability curve for the base-case 2% HBsAg prevalence.

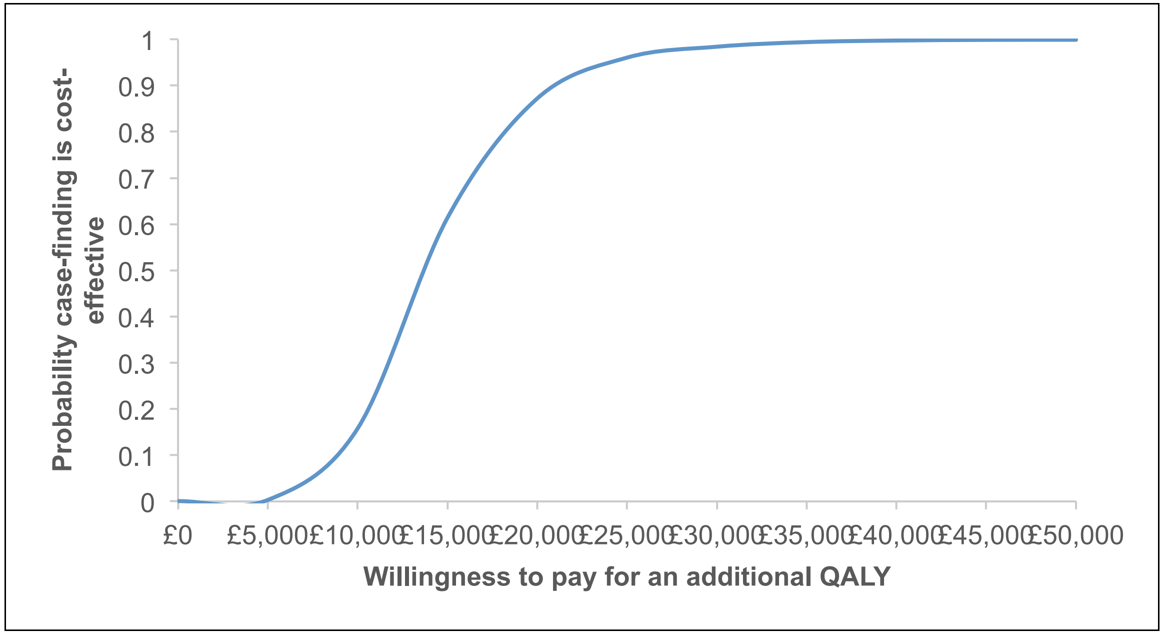

Reference

1. Shepherd J, Jones J, Takeda A, Davidson P, Price A. Adefovir dipivoxil and pegylated interferon alfa-2a for the treatment of chronic hepatitis B: a systematic review and economic evaluation. Health Technology Assessment. 2006;10(28).
2. Marcellin P, Gane E, Buti M, Afdhal N, Sievert W, Jacobson IM, et al. Regression of cirrhosis during treatment with tenofovir disoproxil fumarate for chronic hepatitis B: a 5-year open-label follow-up study. Lancet. 2013 Feb 9;381(9865):468-75. PubMed PMID: 23234725. Epub 2012/12/14. eng.
3. Briggs A, Sculpher M, Claxton K. Decision modelling for health economic evaluation. Gray A, Briggs A, editors. Oxford: Oxford University Press; 2006.
4. Takeda A, Jones J, Shepherd J, Davidson P, Price A. A systematic review and economic evaluation of adefovir dipivoxil and pegylated interferon-alpha-2a for the treatment of chronic hepatitis B. Journal of Viral Hepatitis. 2007;14:75-88.
