# Supplementary material for: Chronic hepatitis B virus case-finding in UK populations born abroad in intermediate or high endemicity countries: an economic evaluation
Source: BMJ Open. 2019 Jun 28;9(6):e030183. doi: 10.1136/bmjopen-2019-030183 (PMC6609059; doi:10.1136/bmjopen-2019-030183)
Supplement: Author's manuscript [file bmjopen-2019-030183.draft_revisions.pdf]

BMJ Open is committed to open peer review. As part of this commitment we make the peer review history of every article we publish publicly available.

When an article is published we post the peer reviewers' comments and the authors' responses online. We also post the versions of the paper that were used during peer review. These are the versions that the peer review comments apply to.

The versions of the paper that follow are the versions that were submitted during the peer review process. They are not the versions of record or the final published versions. They should not be cited or distributed as the published version of this manuscript.

BMJ Open is an open access journal and the full, final, typeset and author-corrected version of record of the manuscript is available on our site with no access controls, subscription charges or pay-per-view fees (http://bmjopen.bmj.com).

If you have any questions on BMJ Open's open peer review process please email info.bmjopen@bmj.com

BMJ Open

Chronic Hepatitis B virus case-finding in populations born abroad in medium or high endemicity countries: an economic evaluation

Journal:	*BMJ Open*
Manuscript ID	bmjopen-2019-030183
Article Type:	Research
Date Submitted by the Author:	04-Mar-2019

Complete List of Authors:	Martin, Natasha; University of California San Diego; University of Bristol, Population Health Sciences<br>Vickerman, Peter; University of Bristol, Population Health Sciences<br>Khakoo, Salim; University of Southampton<br>Ghosh, Anjan; NHS London Borough of Bexley<br>Ramsay, Mary; Public Health England, immunisation; London School of Hygiene and Tropical Medicine, Epidemiology<br>Hickman, M; University of Bristol, Population Health Sciences<br>Williams, Jack; London School of Hygiene and Tropical Medicine<br>Miners, Alec; London School of Hygiene and Tropical Medicine

Keywords:	Hepatology < INTERNAL MEDICINE, hepatitis b virus, HEALTH ECONOMICS, economic evaluation, case-finding, health services research

SCHOLARONE™
Manuscripts

Title: Chronic Hepatitis B virus case-finding in populations born abroad in medium or high endemicity countries: an economic evaluation

Authors: Natasha K Martin[1,2], Peter Vickerman[1], Salim Khakoo[3], Anjan Ghosh[4], Mary Ramsay[5], Matthew Hickman[2], Jack Williams[6] and Alec Miners[6]

Affiliations [1]Division of Infectious Diseases and Global Public Health, University of California San Diego, USA. [2]Population Health Sciences, University of Bristol, UK. [3]Clinical and Experimental Sciences, Faculty of Medicine, University of Southampton, Southampton, UK. [4]NHS London Borough of Bexley, London, UK. [5]Public Health England, London, UK. [6]Department of Health Services Research and Policy, London School of Hygiene and Tropical Medicine, London, UK.

Corresponding author: Dr Alec Miners, 15-17 Tavistock Place, Faculty of Public Health and Policy, London School of Hygiene & Tropical Medicine, London, WC1 9SH, UK

T: 00 44 (0) 0207 927 2069

E: Alec.miners@lshtm.ac.uk

Keywords: Hepatitis B virus, economic evaluation, case-finding and health services research

Abbreviations: HBV: Hepatitis B Virus; HBsAg: hepatitis B virus surface antigen; HBeAg: hepatitis B virus e antigen; ALT: alanine transaminase; DNA: deoxyribose nucleic acid

For peer review only

Financial Support: This work was originally funded by the UK National Institute for Health and Care Excellence. PV, MR and MH acknowledge support from the NIHR Health Protection Research Unit in Evaluation of Interventions at the University of Bristol. NM, PV, and MH acknowledge funding from National Institute for Drug Abuse R01 DA037773. NM also acknowledges funding from the University of California San Diego Center for AIDS Research(CFAR), a National Institute of Health (NIH) funded program [grant number P30 AI036214] , which is supported by the following NIH Institutes and Centers: NIAID, NCI, NIMH, NIDA, NICHD, NHLBI, NIA, NIGMS, and NIDDK. The views in this publication are those of the authors and not necessarily those of the NIHR or the UK Department of Health. AM, PV and JW are members of the NIHR's Sexually Transmitted Infections and Blood Borne Virus Health Protection Research Unit.

Competing interests: NM and PV have received unrestricted research grants from Gilead, outside the submitted work. NM has received honoraria from Gilead and Merck. MH reports personal fees from Gilead, Abbvie, and MSD. AM, SK, AG, MR, JW have no disclosures.

Word Count: 3392 not including references.

Figures: 4

Tables: 1

For peer review only

ABSTRACT

Objectives: The majority (>90%) of new or undiagnosed cases of hepatitis B virus (HBV) in the UK are among individuals born in countries with intermediate or high prevalence levels. We evaluate the cost-effectiveness of increased HBV case-finding among UK migrant populations, based on a one-time opt out case-finding approach in a primary care setting.

Design: Cost-effectiveness evaluation. A decision model based on a Markov approach was built to assess the progression of HBV infection with and without treatment as a result of case-finding. The model parameters, including the cost and effects of case-finding and treatment, were estimated from the literature. All costs were expressed in 2017/18 GBPs and health outcomes as quality-adjusted life-years (QALYs).

Intervention: HCV case-finding among UK migrant populations in a primary care setting compared to no intervention (background testing).

Results: At a 2% hepatitis B surface antigen (HBsAg) prevalence, the case-finding intervention led to a mean incremental cost-effectiveness ratio (ICER) of £13,625 per QALY gained which was 87% and 98% likely of being cost-effective at willingness to pay (WTP) thresholds of £20,000 and £30,000 per additional QALY, respectively. Sensitivity analyses indicated that the intervention would remain cost-effective under a £20,000 WTP threshold as long as HBsAg prevalence among the migrant population is at least 1%. However, the results were sensitive to a number of parameters, especially the time horizon and probability of treatment uptake.

For peer review only

Conclusions: HBV case-finding using a one-time opt out approach in primary care settings is very likely to be cost-effective among UK migrant populations with HBsAg prevalence ≥1% if the WTP for an additional QALY is around £20,000.

Article Summary

- This is a cost-effectiveness evaluation of increased HBV case-finding among UK migrant populations, based on a one-time opt out case-finding approach in a primary care setting.

Strengths and limitations of this study

- Few studies have evaluated the cost-effectiveness of HBV interventions among populations born abroad in medium to high endemicity countries.
- Strengths include numerous sensitivity analyses assessing how cost-effectiveness varies for a range of different prevalences, intervention effect and cost, thus increasing the generalizability of our results to other similar interventions and different settings.
- Limitations include uncertainty in the exact cost or effect of this intervention if scaled up to a national level.

For peer review only

INTRODUCTION

Worldwide, the burden of liver disease continues to rise and remains an urgent public health problem[1]. It is estimated that viral hepatitis is in the top 10 leading causes of mortality globally[2], the majority due to infection with hepatitis B virus (HBV)[3]. Chronic infection with HBV can lead to liver fibrosis, cirrhosis, hepatocellular carcinoma, and death in the absence of treatment. It is estimated that over 5% of the world's population are chronic carriers of HBV[4]. Globally, HBV burden is highest in low-middle income countries in areas such as Sub-Saharan African and East Asia[3]. HBV is spread through exposure to infected blood or body fluids, with the majority of chronic infections acquired perinatally or during childhood[1]. Recently, effective antiviral treatment for HBV has become available which may achieve long-term viral suppression and slow progression of disease[5, 6].

Around 320 cases of acute hepatitis were reported in England in 2015[7]. The prevalence of chronic hepatitis B (CHB) in the UK is estimated to be 0.4% of all adults[8]. It is further estimated that 80% to 90% of newly diagnosed chronic HBV infections are among migrant individuals living in the UK that were born overseas in countries with intermediate or high HBV prevalence (>2%), such as China or Pakistan[8-11]. Although uncertain, it is also likely that a considerable number of people with chronic HBV remain undiagnosed. For example, in one study in Bristol only 12% of migrants born in countries with endemic prevalence >2% had been tested for HBV[9].

Universal screening of pregnant women to identify and immunize neonates exposed to infection is highly cost-effective and under some circumstances cost-saving[12]. The vast majority of European countries already offer universal immunization against hepatitis B, with six exceptions: Denmark, Finland, Iceland, Norway and Sweden. These countries have a very low HBV endemicity and so it is unlikely to be cost-effective to introduce a separate universal HBV vaccination programme[13]. Recent assessments of the cost-effectiveness of universal childhood HBV vaccination suggest that it may be cost-effective if introduced with other vaccines as a component of a hexavalent vaccine – the UK moved to such a product in 2017[13]. Nonetheless, infant vaccination is unlikely to have a great impact on the prevalence of chronic HBV in countries such as the UK because few transmissions are thought to occur once people have entered the country[14]. For these reasons, there remains a critically important role for case-finding activities. However, while studies have shown the cost-effectiveness of one-time screening programs, where a test offer is mailed to migrant individuals identified through a population registry[15], until recently there has not been a published evaluation from a UK perspective. This changed earlier this year when the results of a randomized controlled trial (Hepfree) showed that incentivized screening of HBV and HCV in first and second-generation migrants in a primary care setting was shown to be effective and cost-effective in the UK [16]. However, in contrast to an incentivized screening approach, pilot data from the UK also indicates that an opt-out HBV case-finding approach in primary care settings was also highly effective, and potentially less expensive[17]. Additionally, it was unclear in the previous analysis for the Hepfree trial how much the cost-effectiveness was driven by HCV versus HBV outcomes, and whether the intervention was cost-effective for HBV alone. Further, it is unknown how the cost-

For peer review only

effectiveness of HBV case-finding could vary for a range of prevalences (which likely vary by country of origin), costs, and uptake rates that may occur when the interventions are rolled out across different settings.

The aim of this paper is to evaluate the cost-effectiveness of increased HBV case-finding among UK populations born in high or medium endemicity countries, based on a one-time opt out case-finding approach in primary care settings. Importantly, to increase the generalizability of our results to other similar interventions and different settings, we assess how the cost-effectiveness of HBV case-finding varies for a range of different prevalences, intervention effect and cost.

METHODS

The economic evaluation was undertaken using a Markov approach, where a closed cohort of individuals move between a set of discrete health states, in this instance on an annual basis[18, 19]. A UK National Health Service's cost perspective was used. All costs were displayed in GBP 2017/18 prices and a 40-year time horizon was used. Health outcomes were expressed in terms of Quality-Adjusted Life-Years (QALYs). QALYs and costs were discounted at 3.5% per annum according to UK National Institute for Health and Care Excellence (NICE) recommendations[20]. Uncertainty in the results was examined using deterministic and probabilistic sensitivity analysis (PSA); distributions shown in the tables relate to the PSA analysis. Each PSA consisted of 5,000 runs. HBV transmission was not included in the model as most infections are likely to occur in UK migrant populations before entering the UK[14].

For peer review only

Intervention and target population

A systematic literature review found few studies evaluating HBV case-finding in migrant or other high-risk populations, nor have many studies been published since this review[16, 21]. Our study evaluates the cost-effectiveness of HBV case-finding in the UK for individuals born in countries with intermediate or high prevalence levels. The base case analysis uses the results from an uncontrolled study; Pakistani/British Pakistani people registered at general practices (GPs) in London's East End were written to and invited to 'opt out' of being tested for hepatitis B and C infection. Those who did not opt out were telephoned and asked to attend a clinic for testing[17]. The intervention was designed to increase the likelihood of testing for each infection, assumed in this analysis to occur over the initial model cycle. After this time, the intervention effect was assumed to be zero, with the probability of testing reverting to background levels. The comparator programme or 'no intervention' was defined as the background likelihood of testing through existing routes such as GUM clinics, antenatal clinics or primary care[22].

Model structure

The Markov model was created to represent HBV disease progression and current understanding of policies regarding disease management (Figure 1). The natural history element of the model was largely based on Shepherd et al.[23, 24] The model starts by creating a cohort of people, a proportion of whom are HBsAg+ (HBV prevalence). HBsAg- individuals remain in the model with a general population level of mortality but incurring no HBV-related costs, other than the possibility of being tested for infection. Known HBsAg+ people were assumed to undergo a full viral

For peer review only

profile when initially diagnosed. Acute HBV infection was not included in the model as it is likely that people would have been infected much longer than 6 months ago.

Mutually exclusive stages of chronic hepatitis B (CHB) that were modelled included: HBeAg seroconverted (where ALT levels and HBV DNA are both low), active CHB hepatitis B e-antigen positive (HBeAg+) disease, active CHB hepatitis B e-antigen negative (HBeAg-) disease, and inactive CHB HBeAg- (where ALT levels and HBV DNA are both low). Individuals progressed from CHB to compensated cirrhosis, decompensated cirrhosis (DC), hepatocellular carcinoma (HCC), liver transplant, and post-transplant stages if appropriate drug treatment was not initiated or failed. Due to the severity of the disease and likely presentation, the infection status of all individuals with CHB was assumed to become known if / as soon as they developed DC, HCC or required a liver transplant. Individuals could die from non-HBV related causes from any health state.

Individuals who had raised ALT and HBV (active) levels and who were CHB HBeAg+ were assessed for fibrosis and offered treatment with pegylated interferon for the first year, followed by tenofovir until seroconversion is achieved (as per NICE guidelines[25]) or later stage CHB developed. We assumed successful treatment of active CHB individuals normalized ALT and lowers HBV DNA levels, therefore moving active HBV HBeAg+ individuals to the HBeAg seroconverted stage. Individuals with no evidence of compensated cirrhosis stopped treatment at this time[25]. Individuals with active CHB who were HBeAg- also received pegylated interferon for the first year, followed by tenofovir if they had not developed inactive CHB HBeAg- disease[25]. However, even following the development of inactive

For peer review only

disease, they were assumed to stay on treatment indefinitely to sustain the achieved level of viral suppression[25]. Individuals with evidence of compensated cirrhosis were assumed to remain on tenofovir as long as no further disease progression was recorded, irrespective of e-antigen status[25]. All individuals were assumed to stop treatment on progression to DC or later stages of disease.

Individuals with CHB whose infection status was unknown and those that tested HBsAg+, but declined treatment, were assumed to develop progressive disease according to a set of defined transition probabilities. A different set of transition probabilities were used to define CHB disease progression for those who accepted treatment. As the focus of this analysis is on case-finding, we do not model possible adverse events associated with treatment or treatment resistance.

Model parameters

HBV prevalence among migrant populations to the UK

There is substantial heterogeneity in HBV burden between different migrant populations in the UK depending on their country of origin. Additionally, HBV prevalence among UK migrants may be different compared with their country of origin; a recent UK study of antenatal testing showed the prevalence in migrants was generally less than published estimates for the country of origin, with only Eastern Asia having a higher than expected prevalence[9]. Public Health England (PHE) data on those undergoing routine diagnostic testing suggests that the HBV prevalence among all Asian or British Asian people in the UK is approximately 2%, however these data do not specify country of origin in any further detail[26]. By contrast, the HBV prevalence estimates obtained through targeted studies or antenatal testing

For peer review only

have identified a range of prevalence among UK migrants born in countries with intermediate-high HBV endemicity, such as 17% (Vietnam-born), 7%-10%[27, 28] (China-born), 3-6% (Somalia-born), 1-3% (Pakistan-born), 0.5-1.5% (Bangladesh-born), 0.7% (Poland-born), and 0.5% (India-born)[14, 29-31]. The recent Hepfree trial found a lower prevalence of 1.1%, varying by country of origin, although this included second generation migrants that were born in the UK[16].

Due to the uncertainty in prevalence within populations, and the likely wide variation between populations, in the base case, we assume an HBV prevalence (HBsAg+) of 2%, but explore a range of values (from 0.05-10%) in the sensitivity analysis.

Transition probabilities

Transition probability values, representing the likelihood of moving between health states, for untreated disease stages were based on those reported in a 2006 UK Health Technology Assessment report (Supplementary Tables 1 and 2)[23].

Background testing rate and diagnostic accuracy

The background rate of testing for migrants in the absence of the intervention was estimated using data from PHE, indicating a probability of 2.6% per year[22]. The HBsAg diagnostic test was assumed to be 100% accurate.

Referral and treatment effect

Few studies have quantified the number of people diagnosed with CHB who are subsequently referred on to, and accept, appropriate further clinical investigations for their infection. However, interruptions in the cascade of care post diagnosis are

For peer review only

known to be an issue in the management of CHB and hepatitis C infection both in the UK and elsewhere, particularly in migrant populations[32]. We therefore include a single probability of being referred for specialist care following a HBsAg+ test results, then attending the appointment and starting treatment for those eligible. In the absence of HBV related data, the probability (0.42) was estimated on the basis of 2004-2015 data supplied by Public Health England (personal communication with Public Health England staff) for people who were identified using algorithmic approaches as being Asian and tested HCV RNA positive who then received treatment. However, we consider this parameter to be highly uncertain and undertake sensitivity analysis around it using a wide range of alternative values (10% to 60%).

While a systematic review and meta-analysis of the effects of drug therapy for CHB is available[33], we chose to estimate the impact of antiviral treatment using Marcellin et al[34] as it analysed a much longer follow up period, 5 years rather than 1 year. For HBeAg+ individuals, it was assumed that 20% would e-antigen seroconvert after 1-year of treatment with pegylated interferon and 5.4% a year following treatment with tenofovir. Giving a 40% seroconversion rate at year 5. For HBeAg- individuals, the process was similar, only that by year 5, 84% would develop inactive disease. This was assumed to relate to a 75% probability of response following the initial 1-year of pegylated interferon and 2.3% a year following treatment with tenofovir. Irrespective of whether individuals were HBeAg+ or HBeAg-, they were assumed to continue treatment after year 5 with tenofovir until they responded to it assuming the same constant rate of response.

For peer review only

The probability of responding to treatment was assumed to be the same for people with or without compensated disease. However, once people developed compensated disease, it was assumed not to regress following treatment, and the costs and disutility associated with it would remain. The only benefit of treatment in this group was slower progression to poorer health states compared with not being treated.

Intervention effect

The base case probability of testing for HBsAg in the intervention arm was based on a one-time ‘opt out’ option within a general practice setting; 223 out of 1,134 (19.7%) eligible tested after being identified using a GP registries database and responding to a written invite[17].

Cohort demographics and initial stage distribution

PHE data suggests that the average age at HBV diagnosis in the UK Asian population is approximately 35 years of age[26], which we use as the base-case starting age in our model but vary in the sensitivity analysis. The proportion of people with CHB who were, or were originally, HBeAg+, rather than HBeAg-, was assumed to be 0.14 ([71/490] personal communication with Public Health England staff). The proportion of people who had subsequently seroconverted, or developed inactive disease, before being tested for HBsAg, was assumed to be 80% (personal communication with Public Health England staff). It was further assumed that 44% of people with active HBeAg+ or HBeAg- disease, had already developed compensated cirrhosis[35].

For peer review only

Health utilities and costs

Utility values related to HBV infection were sourced from the review by Shepherd et al[23] and Takeda et al[24] (Supplementary Table 3). The costs of HBV testing/monitoring, antiviral treatment, and health-state specific costs were taken from a number of published sources[23, 35] (Table 1), inflated to GBP £2017 where appropriate using the NHS Hospital and Community Health Services Pay and Prices Index and the Health Service Cost Index[36, 37]. The intervention cost was estimated at £4 per person eligible for testing. This cost relates to the resources required to identify and invite each individual for a test and excludes the cost of any tests and treatments. Thus, if 100 individuals were eligible for testing, the total cost of the intervention was £400 irrespective of how many people attended for a test. The importance of this assumption was assessed in the sensitivity analysis given the extent of uncertainty.

Sensitivity analyses

To test the robustness of the results to alternative assumptions, we have undertaken extensive one-way sensitivity analyses. The results of a probabilistic sensitivity analysis (PSA) are also reported, in which relevant parameters are simultaneously sampled 5,000 times. Finally, due to the uncertainty surrounding the intervention cost and impact if scaled-up to the national level and among different migrant populations, we undertake a threshold analysis where we evaluate the HBV prevalence at which the intervention is cost-effective at a willingness to pay (WTP) ICER threshold of <£20,000 per additional QALY with varying intervention cost (between £1 and £20, £4 per person eligible at base-case), intervention effect

For peer review only

(between 5% and 30%, 19.7% uptake at base-case) and HBsAg prevalence (between 1% and 10%, 2% base-case) – the results are displayed as a contour map.

RESULTS

Base-case 2% HBsAg prevalence

At a 2% HBsAg prevalence, the case-finding intervention resulted in mean incremental costs and QALYs of about £28 and 0.002 respectively over the 5,000 samples, corresponding to an ICER of £13,625 per QALY gained (95% credible interval £7,121 to £27,588). The intervention was 87% and 98% likely to be cost-effective at £20,000 and £30,000 WTP per additional QALY thresholds, respectively (Supplementary Figure 1). Most of the univariate sensitivity analyses produced ICERs below a £20,000 WTP threshold (Figure 2), including reducing the likelihood of testing from 19.7% to 5% (£19,323 / QALY gained). However, the exceptions were assuming a 20-year time horizon instead of 40 years (£22,713 / QALY gained), discounting QALYs at 6% instead of 3.5% (£21,970 / QALY gained), not discounting costs instead of 6% (21,521 / QALY gained) and doubling the costs of all drug treatments from £3,979 / £2,453 to £7,957 / £4,905 (£22,586 / QALY gained). Decreasing the probability of treatment uptake after testing positive for HBsAg from 0.42 to 0.1 increased the ICER to over £30,000 (£31,340 / QALY gained).

Impact of variation in HBV prevalence and intervention impact (cost, effect and uptake)

Cost-effectiveness is strongly driven by HBV prevalence. Our sensitivity analyses indicated that the intervention would remain cost-effective under a £20,000 WTP

For peer review only

threshold as long as HBV prevalence among the migrant population is equal to or exceeds 1% (Figure 3).

Due to the uncertainty in cost and intervention impact if scaled-up across the UK and among different migrant population, we additionally present a sensitivity analysis of the threshold HBV prevalence which would ensure the intervention is cost-effective under a £20,000 WTP with varying costs and intervention effects (Figure 4). The contour map shows that, for example, the intervention would be cost-effective at a prevalence of 1% if it cost £6 per person and the intervention effect was 20%. However, it would no longer be cost-effective at a 1% prevalence level and £6 cost if the intervention effect reduced to 10%.

DISCUSSION

HBV case-finding using a one-time opt out approach in primary care settings has a high potential to be cost-effective among UK migrant populations with a HBV prevalence at or above an average of 1%. However, the results are sensitive to a number of factors including the intervention effect or cost, rate of treatment uptake, assuming a much shorter time horizon and (unrealistically) high discount rates and drug costs.

Limitations

The main limitation with the analysis is the substantial uncertainty surrounding the costs of the intervention and its effect if this case-finding intervention were scaled-up to a national level. Nonetheless, extensive sensitivity analysis shows that the intervention remained cost-effective across a large range of evaluated scenarios.

For peer review only

Thus, while establishing more robust estimates of the costs and effects of interventions to find cases of HBV will undoubtedly decrease the uncertainty around our results, we believe the scope for the modelled intervention to be cost-effective is extremely high.

Current UK NHS HBV-testing policy is to contact household members once a case has been identified. However, we were unable to include this aspect in our analysis due to a lack of data specific to the target migrant populations on the size and age distribution of households of infected contacts, the probability that contacts were HBsAg+ and the likelihood that contacts could be traced in the first instance. The impact of excluding this process on the ICER we report is difficult to determine. For example, if contact tracing results in a high proportion of people being treated for CHB the ICER could decrease. Conversely, if many HBsAg- people are vaccinated against HBV, the ICER could increase as there is already evidence to suggest it is unlikely to be cost-effective[13].

Finally, we did not model the possibility of simultaneously testing for hepatitis C virus (HCV), which may increase the cost-effectiveness of the intervention though evidence on the HCV prevalence among migrants also has uncertainies[19].

Comparison with other studies

Five studies have examined the cost-effectiveness of screening for HBV among migrant populations. A Dutch study[15] found that screening migrants from countries with high or intermediate HBV prevalence (assuming a 3.4% chronic infection prevalence) was highly cost-effective (EUR9000 per QALY gained) at a screening

campaign cost of approximately EUR11 per person eligible and 35% uptake – which is consistent with our sensitivity analysis. Another study explored screening and treatment of migrants from Asian and Pacific Islands in the US[38], finding it to be cost-effective (US$36,000 per QALY gained) but also assuming a much higher prevalence of HBV (10%), screening uptake (70%) and no screening programme costs aside from the diagnostic tests. Two studies examined the cost-effectiveness of screening all migrants to Canada[39, 40], both finding tenofovir-based treatment moderately cost-effective (CAD$40,000/QALY [~£22,000]) at 4.8-6.5% chronic infection prevalence's. Our model assumes a lower prevalence of chronic HBV, higher treatment efficacy and lower treatment and screening costs than the North American studies, which may explain the difference in cost-effectiveness estimates. Lastly, our results are partially consistent with findings from the recent Hepfree trial, which was found to be cost-effective (£8,540/QALY) for a similar observed intervention effect (19.7% uptake of testing compared to 19.5% uptake in our study). However, Hepfree had higher intervention costs (>£25 per patient compared to £1-20 in our model), combined HCV and HBV screening and identified patients on basis of ethnic group rather than country of birth [41].

Conclusions

Our analysis suggests that interventions to increase HBV case-finding in primary care among UK migrant populations with a prevalence of at least 1% – such as using a one-time opt out approach – could be cost-effective – underpinning current National Institute for Health and Care Excellence guidance[42]. Critically, at a threshold prevalence above 1% this will encompass migrant populations from most countries with endemic HBV, even if there is a healthy migrant effect (with migrant

For peer review only

populations in UK on average at lower risk than people in their country of origin[14]). These recent results support the recommendation that interventions to increase HBV case-finding in primary care among UK migrant populations should be expanded, but needs to be based on screening by country of birth rather than ethnic group.

For peer review only

Author contributions: AM, PV, MH designed the study. AM, AG, JW and NM coded the analysis. All authors interpreted the data. AM and NM wrote the first draft. All authors contributed to the manuscript drafting, approved of the final version, and agreed to authorship.

Data Sharing: Model code available on request to the corresponding author.

License statement: I, the Submitting Author has the right to grant and does grant on behalf of all authors of the Work (as defined in the below author licence), an exclusive licence and/or a non-exclusive licence for contributions from authors who are: i) UK Crown employees; ii) where BMJ has agreed a CC-BY licence shall apply, and/or iii) in accordance with the terms applicable for US Federal Government officers or employees acting as part of their official duties; on a worldwide, perpetual, irrevocable, royalty-free basis to BMJ Publishing Group Ltd ("BMJ") its licensees and where the relevant Journal is co-owned by BMJ to the co-owners of the Journal, to publish the Work in BMJ Open and any other BMJ products and to exploit all rights, as set out in our licence.

The Submitting Author accepts and understands that any supply made under these terms is made by BMJ to the Submitting Author unless you are acting as an employee on behalf of your employer or a postgraduate student of an affiliated institution which is paying any applicable article publishing charge ("APC") for Open Access articles. Where the Submitting Author wishes to make the Work available on an Open Access basis (and intends to pay the relevant APC), the terms of reuse of

For peer review only

such Open Access shall be governed by a Creative Commons licence – details of these licences and which Creative Commonslicence will apply to this Work are set out in our licence referred to above.

Patient and Public Involvement: This study was commissioned by the UK National Institute for Health and Care Excellence (https://www.nice.org.uk/guidance/ph43/) with representation from lay members on the guidance panel who contributed to shaping the proposed intervention and interpretation of the study findings and implications.

For peer review only

1

For peer review only

FIGURE LEGENDS

Figure 1. HBV model schematic. The arrows denote possible transitions between states; HBsAg, hepatitis B virus surface antigen; HBeAg: hepatitis b virus e antigen; CHB, chronic hepatitis B virus; CC, compensated cirrhosis; DC, decompensated cirrhosis; HCC, hepatocellular carcinoma; LT, liver transplant; *individuals may or may not know their infection status; %individuals with CC responding to treatment were assumed to keep the costs and utility associated with CC, but with disease progression probabilities equivalent to HBeAg seroconversion / inactive disease; “transitions permitted from all health states to death

Figure 2: Univariate sensitivity analysis on the ICER with a 2% HBV prevalence scenario. ICER, incremental cost-effectiveness ratio; Y-axis indicates the base case ICER of £21,400 per QALY gained; *halves or doubles all baseline drug costs where relevant

Figure 3. Mean incremental cost-effectiveness ratio (ICER) of HBV screening by varying HBsAg prevalence

Figure 4. Contour map showing for a range of costs (horizontal axis) and intervention effects (vertical axis), the threshold HBV prevalence (contours) where the intervention ICER falls under a £20,000 willingness to pay threshold.

For peer review only

Table 1: Annual costs in 2017/18 UK prices (£)

Cost	Mean	95% interval of sampled range^	Source
**Intervention cost per person eligible for testing***	4	-	Assumption
**HBsAg test (laboratory)**	10	-	Assumption
**Pegylated interferon**	3,979	-	BNF[43]
**Tenofovir**	2,453	-	BNF[43]
**ALT and ultrasound**	77	-	Assumption[43]
**Full viral profile**	432	-	Assumption[43]
**HBeAg+ seroconverted / HBeAg- ALT/DNA low[a]**	335	240-446	Shepherd[23]
**HBeAg+ / HBeAg- active disease[b]**	674	480-896	Shepherd[23]
**Compensated cirrhosis**	1,606	1,052-2,283	Crossan[35]
**Decompensated cirrhosis**	38,212	21,848-60,645	Crossan[35]
**Hepatocellular carcinoma**	38,212	21,848-60,645	Crossan[35]
**Liver transplant (first year)**	67,698	57,301-79,287	Crossan[35]
**Liver transplant (subsequent years)**	17,231	5,415-35,399	Crossan[35]

*Indicates a one off cost; ^Sampled values from the probabilistic sensitivity analysis using a gamma distribution; [b]costs are additional to[a]

For peer review only

References

1. World Health Organisation. *Global hepatitis report, 2017*. 2017 04/08/2017]; Available from: http://apps.who.int/iris/bitstream/10665/255016/1/9789241565455-eng.pdf?ua=1.
2. Cooke, G.S., M. Lemoine, M. Thursz, C. Gore, T. Swan, A. Kamarulzaman, et al., *Viral hepatitis and the Global Burden of Disease: a need to regroup.* J Viral Hepat, 2013. **20**(9): p. 600-1.
3. Lozano, R., M. Naghavi, K. Foreman, S. Lim, K. Shibuya, V. Aboyans, et al., *Global and regional mortality from 235 causes of death for 20 age groups in 1990 and 2010: a systematic analysis for the Global Burden of Disease Study 2010.* The Lancet, 2012. **380**(9859): p. 2095-2128.
4. World Health Organisation, *Hepatitis B fact sheet*. 2014.
5. Rajbhandari, R. and R.T. Chung, *Treatment of Hepatitis B: A Concise Review.* Clinical and Translational Gastroenterology, 2016. **7**(9): p. e190.
6. World health Organisation. *Guidelines for the prevention, care and treatment of persons with chronic hepatitis B infection*. Guidelines 2015 16/06/2017]; Available from: http://apps.who.int/iris/bitstream/10665/154590/1/9789241549059_eng.pdf?ua=1&ua=1.
7. Public Health England, *Acute hepatitis B (England): annual report 2017*. 2018.
8. Health Protection Agency, *Migrant Health: Infectious diseases in non-UK born populations in the UK. An update to the baseline report*. 2011.
9. Cochrane, A., I. Evlampidou, C. Irish, S.M. Ingle, and M. Hickman, *Hepatitis B infection prevalence by country of birth in migrant populations in a large UK city.* J Clin Virol, 2015. **68**: p. 79-82.
10. Hahne, S., M. Ramsay, K. Balogun, W.J. Edmunds, and P. Mortimer, *Incidence and routes of transmission of hepatitis B virus in England and Wales, 1995-2000: implications for immunisation policy.* J Clin Virol, 2004. **29**(4): p. 211-20.
11. Evlampidou, I., M. Hickman, C. Irish, N. Young, I. Oliver, S. Gillett, et al., *Low hepatitis B testing among migrants: a cross-sectional study in a UK city.* British Journal of General Practice, 2016.
12. Edmunds, W.J. and R. Ramsay, *The estimated cost-effectiveness of vcaccination in infants born to hepatitis B positive mothers*, in *NICE Public Health Guidance no. 21*. 2009.
13. Siddiqui, M.R., N. Gay, W.J. Edmunds, and M. Ramsay, *Economic evaluation of infant and adolescent hepatitis B vaccination in the UK.* Vaccine, 2011. **29**(3): p. 466-475.
14. Uddin, G., D. Shoeb, S. Solaiman, R. Marley, C. Gore, M. Ramsay, et al., *Prevalence of chronic viral hepatitis in people of south Asian ethnicity living in England: the prevalence cannot necessarily be predicted from the prevalence in the country of origin.* Journal of Viral Hepatitis, 2010. **17**: p. 327-335.
15. Veldhuijzen, I.K., M. Toy, S.J.M. Hahné, G.A. De Wit, S.W. Schalm, R.A. de Man, et al., *Screening and Early Treatment of Migrants for Chronic Hepatitis B Virus Infection Is Cost-Effective.* Gastroenterology, 2010. **138**(2): p. 522-530.

For peer review only

16. Flanagan, S., J. Kunkel, V. Appleby, S.E. Eldridge, S. Ismail, S. Moreea, et al., *Case finding and therapy for chronic viral hepatitis in primary care (HepFREE): a cluster-randomised controlled trial.* Lancet Gastroenterol Hepatol, 2019. **4**(1): p. 32-44.
17. Lewis, H., K. Burke, S. Begum, I. Ushiro-Limb, and G.R. Foster, *What is the best method of case finding for chronic viral hepatitis in migrant communities?* Gut, 2011. **Vol 60 Suppl 2**: p. A26.
18. Beck, J.R. and S.G. Pauker, *The Markov process in medical prognosis.* Medical Decision Making, 1983. **3**(4): p. 419-458.
19. Sonnenberg, F.A. and J.R. Beck, *Markov models in medical decision making: a practical guide.* Medical Decision Making, 1993. **13**(4): p. 322-338.
20. National Institute for Health and Care Excellence, *Guide to the methods of technology appraisal*. 2013.
21. Jones, L., G. Bates, E. McCoy, C. Beynon, J. McVeigh, and M. Bellis. *A systematic review of the effectiveness & cost-effectiveness of interventions aimed at raising awareness and engaging with groups who are at an increased risk of hepatitis B and C infection*. 2011 23/03/2017]; Available from: https://www.nice.org.uk/guidance/ph43/evidence/evidence-review-2-69062510.
22. Health Protection Agency, *Sentinel Surveillance of Hepatitis Testing in England - Hepatitis B and D 2010 Report - Analysis of testing between 2007 and 2010*. 2011: Collindale, UK.
23. Shepherd, J., J. Jones, A. Takeda, P. Davidson, and A. Price, *Adefovir dipivoxil and pegylated interferon alfa-2a for the treatment of chronic hepatitis B: a systematic review and economic evaluation.* Health Technology Assessment, 2006. **10**(28).
24. Takeda, A., J. Jones, J. Shepherd, P. Davidson, and A. Price, *A systematic review and economic evaluation of adefovir dipivoxil and pegylated interferon-alpha-2a for the treatment of chronic hepatitis B.* Journal of Viral Hepatitis, 2007. **14**: p. 75-88.
25. National Institute for Health and Care Excellence. *Hepatitis B (chronic): diagnosis and management*. 2013 24/03/2017]; Available from: https://www.nice.org.uk/guidance/Cg165.
26. Public Health England. *Annual report from the sentinel surveillance study of blood borne virus testing in England: data for January to December 2015*. Health protection report 2016 02/06/2017]; Available from: https://www.gov.uk/government/uploads/system/uploads/attachment_data/file/540332/hpr2416_bbvs.pdf.
27. McPherson, S., M. Valappil, S. Moses, G. Eltringham, C. Miller, K. Baxter, et al., *CHASE-B (Chinese hepatitis awareness, surveillance, and education): A pilot of targeted case finding for hepatitis B virus (HBV) in the British-Chinese community.* Gut, 2010. **60**: p. A25-A26.
28. Kawsar, M.T. and B.T. Goh, *Hepatitis B virus infection among Chinese residents in the United Kingdom.* Sexually Transmitted Infections, 2002. **78**: p. 166-168.

For peer review only

29. Aweis, D., B.J. Brabin, N.J. Beeching, J.E. Bunn, C. Cooper, K. Gardner, et al., *Hepatitis B prevalence and risk factors for HBsAg carriage amongst Somali households in Liverpool.* Commun Dis Public Health, 2001. **4**(4): p. 247-52.
30. Brabin, B., N.J. Beeching, J.E. Bunn, C. Cooper, K. Gardner, and C.A. Hart, *Hepatitis B prevalence among Somali households in Liverpool.* Arch Dis Child, 2002. **86**(1): p. 67-8.
31. McPherson, S., M. Valappil, S.E. Moses, G. Eltringham, C. Miller, K. Baxter, et al., *Targeted case finding for hepatitis B using dry blood spot testing in the British-Chinese and South Asian populations of the North-East of England.* J Viral Hepat, 2013. **20**(9): p. 638-44.
32. Vedio, A., E.Z.H. Liu, A.C.K. Lee, and S. Salway, *Improving access to health care for chronic hepatitis B among migrant Chinese populations: A systematic mixed methods review of barriers and enablers.* Journal of Viral Hepatitis, 2017. **24**(7): p. 526-540.
33. Woo, G., G. Tomlinson, Y. Nishikawa, M. Kowgier, M. Sherman, D.K. Wong, et al., *Tenofovir and entecavir are the most effective antiviral agents for chronic hepatitis B: a systematic review and Bayesian meta-analyses.* Gastroenterology, 2010. **139**(4): p. 1218-1229.
34. Marcellin, P., E. Gane, M. Buti, N. Afdhal, W. Sievert, I.M. Jacobson, et al., *Regression of cirrhosis during treatment with tenofovir disoproxil fumarate for chronic hepatitis B: a 5-year open-label follow-up study.* Lancet, 2013. **381**(9865): p. 468-75.
35. Crossan, C., E.A. Tsochatzis, L. Longworth, K. Gurusamy, B. Davidson, M. Rodriguez-Peralvarez, et al., *Cost-effectiveness of non-invasive methods for assessment and monitoring of liver fibrosis and cirrhosis in patients with chronic liver disease: systematic review and economic evaluation.* Health Technol Assess, 2015. **19**(9): p. 1-409, v-vi.
36. Curtis, L. and A. Burns. *Unit costs of health and social care 2015*. 2015 27/03/2017]; Available from: http://www.pssru.ac.uk/project-pages/unit-costs/2015/.
37. Curtis, L. and A. Burns. *Unit costs of health and social care 2018*. 2018 18/12/2018]; Available from: https://www.pssru.ac.uk/pub/uc/uc2018/sources-of-information.pdf.
38. Hutton, D.W., D. Tan, S.K. So, and M.L. Brandeau, *Cost-effectiveness of screening and vaccinating Asian and Pacific Islander adults for hepatitis B.* Ann Intern Med, 2007. **147**(7): p. 460-9.
39. Rossi, C., K. Schwartzman, O. Oxlade, M.B. Klein, and C. Greenaway, *Hepatitis B screening and vaccination strategies for newly arrived adult Canadian immigrants and refugees: a cost-effectiveness analysis.* PLoS One, 2013. **8**(10): p. e78548.
40. Wong, W.W.L., G. Woo, E. Jenny Heathcote, and M. Krahn, *Cost effectiveness of screening immigrants for hepatitis B.* Liver International, 2011. **31**(8): p. 1179-1190.
41. Hickman, M., S. Mandel, P. Vickerman, A. Miners, and N. Martin, *Hepatitis case finding among migrants in primary care.* Lancet Gastroenterol Hepatol, 2019. **4**(1): p. 3-4.

For peer review only

42. National Institute for Health and Clinical Excellence. *Hepatitis B and C - ways to promote and offer testing*. 2012; Available from: http://publications.nice.org.uk/hepatitis-b-and-c-ways-to-promote-and-offer-testing-to-people-at-increased-risk-of-infection-ph43.
43. Royal Pharmaceutical Society. *British National Formulary no. 71*. 2016 01/08/2016]; Available from: www.bnf.org.
44. Briggs, A., M. Sculpher, and K. Claxton, *Decision modelling for health economic evaluation*. Handbooks in health economic evaluation series, ed. A. Gray and A. Briggs. 2006, Oxford: Oxford University Press.

For peer review only

For peer review only

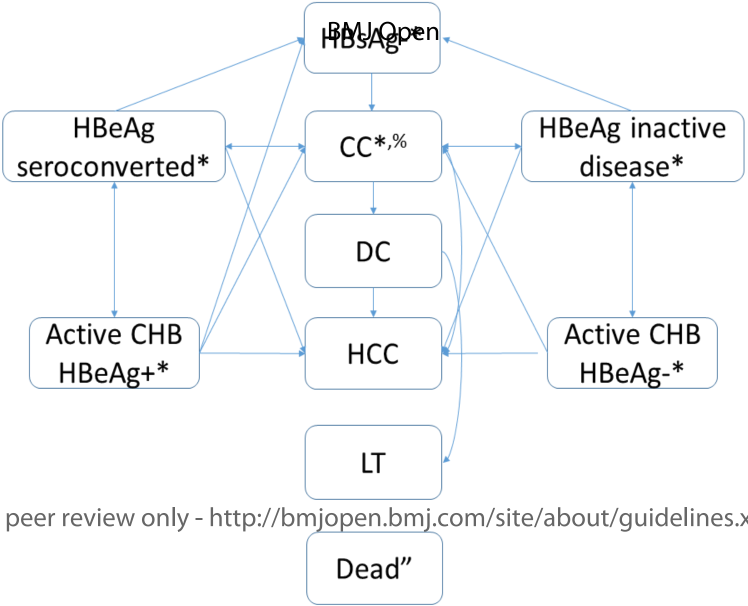

For peer review only - http://bmjopen.bmj.com/site/about/guidelines.xhtml

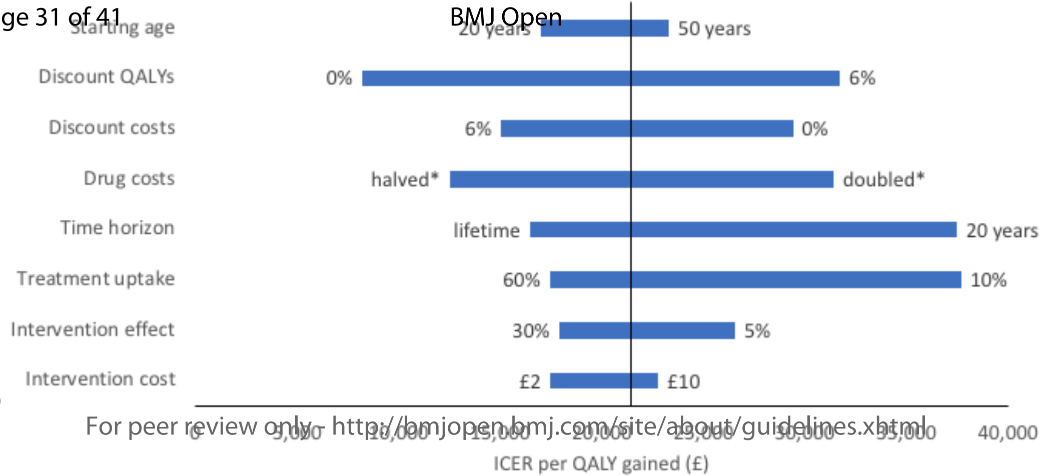

For peer review only - http://bmjopen.bmj.com/site/about/guidelines.xhtml

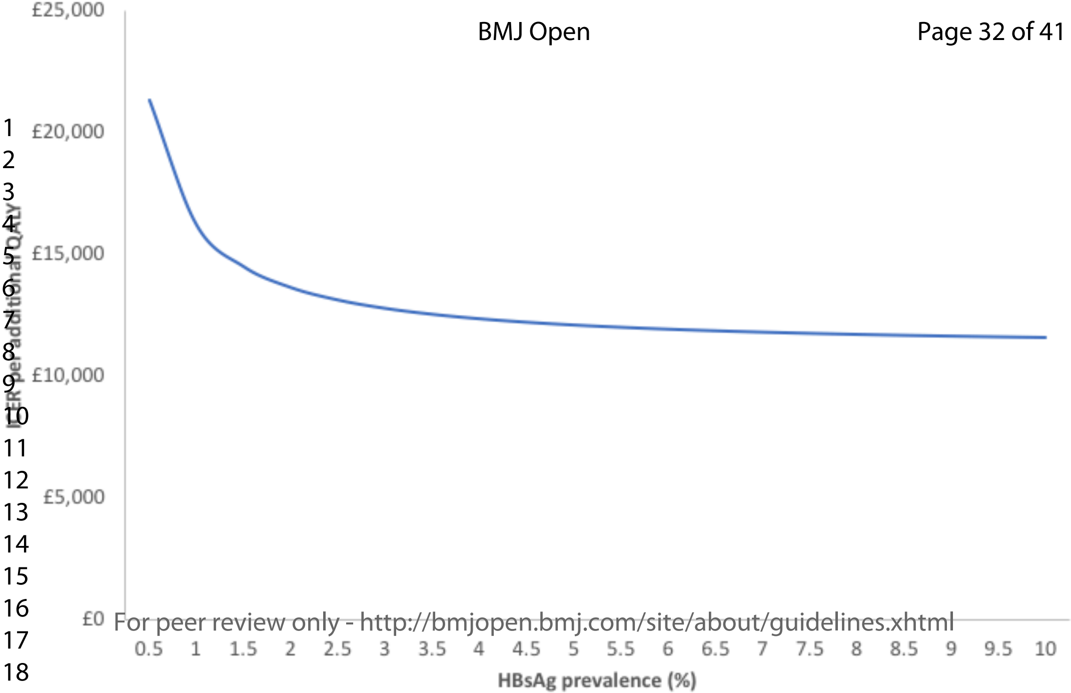

For peer review only - http://bmjopen.bmj.com/site/about/guidelines.xhtml

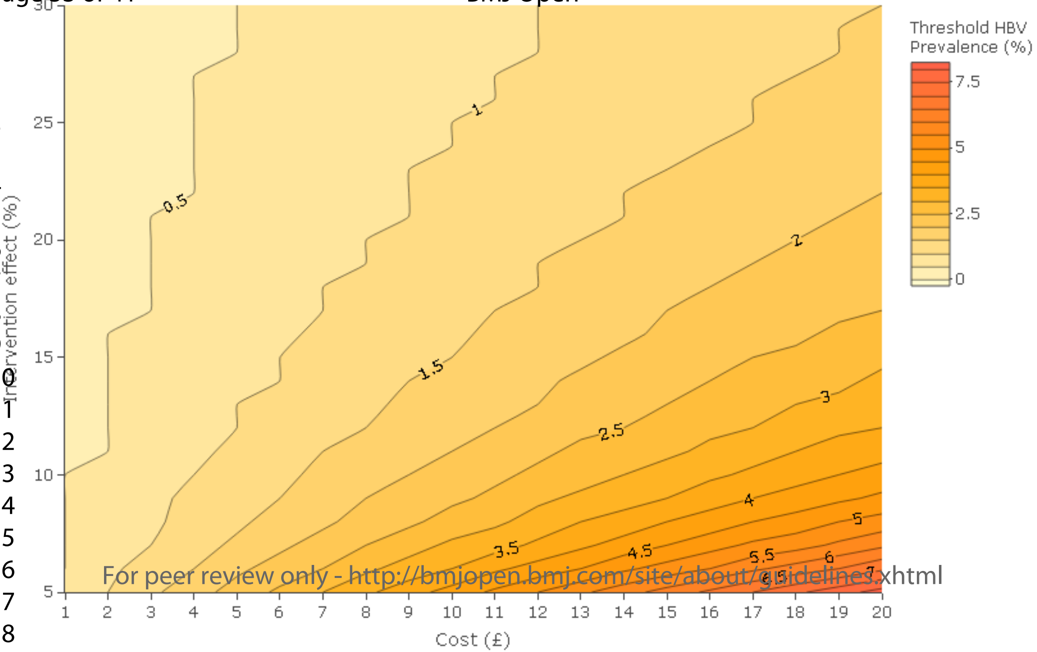

For peer review only - http://bmjopen.bmj.com/site/about/guidelines.xhtml

Supplementary Materials

Supplementary Table 1: Annual transition probability matrix for people who enter the model as HBsAg+ and HBeAg+ derived from Shepherd(1) and Marcellin(2)

**To:** / **From:**	**HBsAg seroconverted**	**HBeAg seroconverted**	**CHB HBeAg+ active disease**	**CC**	**DC**	**HCC**	**LT1**	**LT2**	**Dead⁺**
**HBsAg seroconverted**	#	0	0	0	0	0.00005	0	0	0
**HBeAg seroconverted**	0.02	#	0.03	0.01	0	0.005	0	0	0
**CHB HBeAg+ active disease no treatment**	0.0175	0.05	#	0.05	0	0.005	0	0	0.0035
**CHB HBeAg+ active disease or CC on treatment**									
**Treatment response with peglyated interferon**	0.0175	0.20	#	0.05	0	0.005	0	0	0.0035
**Treatment response with tenofovir**	0.0175	0.054	#	0.05	0	0.005	0	0	0.0035
**Compensated cirrhosis (CC) no treatment**	0	0.05	0	#	0.05	0.025	0	0	0.051
**Decompensated cirrhosis (DC)**	0	0	0	0	#	0.025	0.03	0	0.39
**Hepatocellular carcinoma (HCC)**	0	0	0	0	0	#	0	0	0.56
**Liver transplant – first year (LT1)**	0	0	0	0	0	0	#	0	0.21
**Liver transplant – subsequent years (LT2)**	0	0	0	0	0	0	0	#	0.057

⁺an age-adjusted general population mortality is added to this amount; #, indicates the residual row probability; all values are converted to a Dirichlet distribution by assuming an effective sample size of 200 for each row(3)

For peer review only

Supplementary Table 2: Annual transition probability matrix for people who enter the model as HBsAg+ and HBeAg- derived from Shepherd(1) and Marcellin(2)

To: / From:	HBsAg seroconverted	HBeAg seroconverted	CHB HBeAg- active disease	CC	DC	HCC	LT1	LT2	Dead[+]
**HBsAg seroconverted**	#	0	0	0	0	0.00005	0	0	0
**HBeAg seroconverted**	0.0175	#	0.03	0.01	0	0.005	0	0	0
**CHB HBeA- active disease no treatment**	0	0.015	#	0.05	0	0.005	0	0	0.0035
**CHB HBeAg- active disease or CC on treatment**									
**Treatment response with peglyated interferon**	0	0.75	#	0.05	0	0.005	0	0	0.0035
**Treatment response with tenofovir**	0	0.023	#	0.05	0	0.005	0	0	0.0035
**Decompensated cirrhosis (DC)**	0	0	0	0	#	0.025	0.03	0	0.39
**Hepatocellular carcinoma (HCC)**	0	0	0	0	0	#	0	0	0.56
**Liver transplant – first year (LT1)**	0	0	0	0	0	0	#	0	0.21
**Liver transplant – subsequent years (LT2)**	0	0	0	0	0	0	0	#	0.057

[+]an age-adjusted general population mortality is added to this amount; #, indicates the residual row probability; all values are converted to a Dirichlet distribution by assuming an effective sample size of 200 for each row(3)

For peer review only

Supplementary Table 3: Utility values from Shepherd(1) and Takeda(4)

Utility	Mean decrement	95% interval of sampled range^
**Age**		-
**0-44**	0.09	-
**45-54**	0.15	-
**55-64**	0.20	-
**65-74**	0.22	-
**75+**	0.27	-
**HBsAg-**	0	-
**HBeAg+ seroconverted / HBeAg- ALT/DNA low**	0	-
**HBeAg+ / HBeAg- active disease**	0.04	0.023-0.062
**Compensated cirrhosis**	0.44	0.34-0.55
**Decompensated cirrhosis**	0.54	0.43-0.73
**Hepatocellular carcinoma**	0.54	0.43-0.73
**Liver transplant (first year)**	0.54	0.43-0.73
**Liver transplant (subsequent years)**	0.32	0.22-0.43

Utility values are calculated by subtracting appropriate decrements from 1; ^Sampled values from the probabilistic sensitivity analysis using a beta distribution

For peer review only

Supplementary Figure 1. Cost-effectiveness acceptability curve for the base-case 2% HBsAg prevalence.

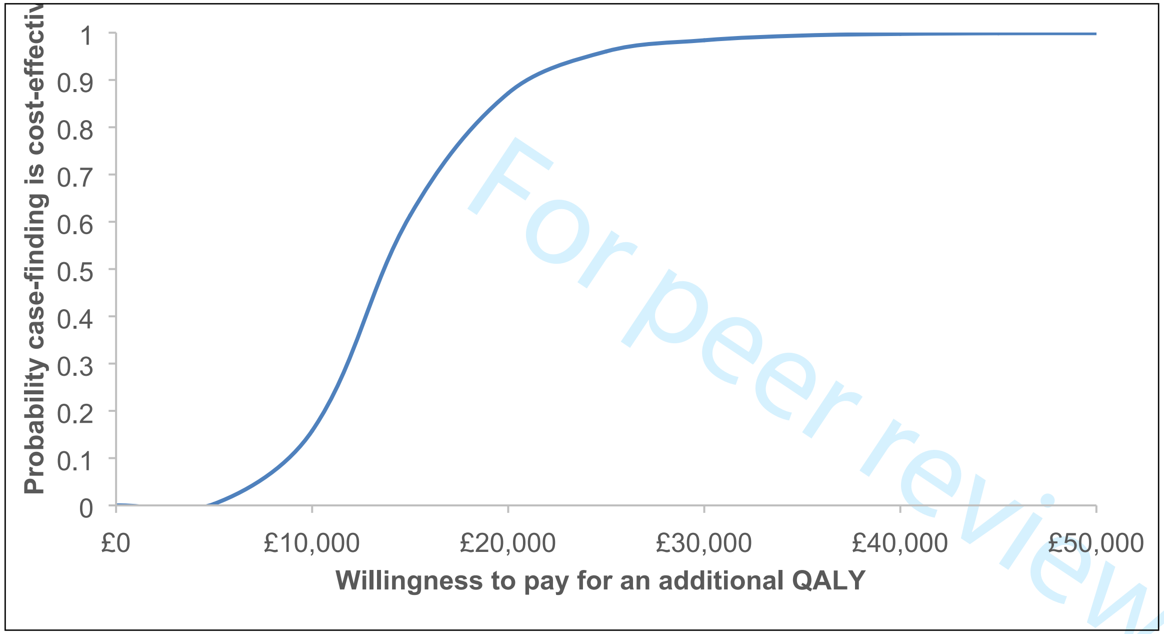

For peer review only

Reference

1. Shepherd J, Jones J, Takeda A, Davidson P, Price A. Adefovir dipivoxil and pegylated interferon alfa-2a for the treatment of chronic hepatitis B: a systematic review and economic evaluation. Health Technology Assessment. 2006;10(28).

2. Marcellin P, Gane E, Buti M, Afdhal N, Sievert W, Jacobson IM, et al. Regression of cirrhosis during treatment with tenofovir disoproxil fumarate for chronic hepatitis B: a 5-year open-label follow-up study. Lancet. 2013 Feb 9;381(9865):468-75. PubMed PMID: 23234725. Epub 2012/12/14. eng.

3. Briggs A, Sculpher M, Claxton K. Decision modelling for health economic evaluation. Gray A, Briggs A, editors. Oxford: Oxford University Press; 2006.

4. Takeda A, Jones J, Shepherd J, Davidson P, Price A. A systematic review and economic evaluation of adefovir dipivoxil and pegylated interferon-alpha-2a for the treatment of chronic hepatitis B. Journal of Viral Hepatitis. 2007;14:75-88.

For peer review only

CHEERS Checklist
Items to include when reporting economic evaluations of health interventions

The **ISPOR CHEERS Task Force Report**, *Consolidated Health Economic Evaluation Reporting Standards (CHEERS)—Explanation and Elaboration: A Report of the ISPOR Health Economic Evaluations Publication Guidelines Good Reporting Practices Task Force*, provides examples and further discussion of the 24-item CHEERS Checklist and the CHEERS Statement. It may be accessed via the *Value in Health* or via the ISPOR Health Economic Evaluation Publication Guidelines – CHEERS: Good Reporting Practices webpage: http://www.ispor.org/TaskForces/EconomicPubGuidelines.asp

Section/item	Item No	Recommendation	Reported on page No/ line No
**Title and abstract**			
Title	1	Identify the study as an economic evaluation or use more specific terms such as "cost-effectiveness analysis", and describe the interventions compared.	Pg 1/Line 1-2
Abstract	2	Provide a structured summary of objectives, perspective, setting, methods (including study design and inputs), results (including base case and uncertainty analyses), and conclusions.	Pg 3/Line 1-25
**Introduction**			
Background and objectives	3	Provide an explicit statement of the broader context for the study. Present the study question and its relevance for health policy or practice decisions.	Pg 6/Line 5-10
**Methods**			
Target population and subgroups	4	Describe characteristics of the base case population and subgroups analysed, including why they were chosen.	Pg 8/lines 4-6
Setting and location	5	State relevant aspects of the system(s) in which the decision(s) need(s) to be made.	Pg 8/lines 4-6
Study perspective	6	Describe the perspective of the study and relate this to the costs being evaluated.	Pg 7/line 16
Comparators	7	Describe the interventions or strategies being compared and state why they were chosen.	Pg 8/lines4-15
Time horizon	8	State the time horizon(s) over which costs and consequences are being evaluated and say why appropriate.	Pg 7/line 17
Discount rate	9	Report the choice of discount rate(s) used for costs and outcomes and say why appropriate.	Pg 7/line 19
Choice of health outcomes	10	Describe what outcomes were used as the measure(s) of benefit in the evaluation and their relevance for the type of analysis performed.	pg 7/lines 18-19
Measurement of effectiveness	11a	*Single study-based estimates:* Describe fully the design features of the single effectiveness study and why the single study was a sufficient source of clinical effectiveness data.	pg 13, lines 8-12

For peer review only

	11b	*Synthesis-based estimates:* Describe fully the methods used for identification of included studies and synthesis of clinical effectiveness data.	n/a
Measurement and valuation of preference based outcomes	12	If applicable, describe the population and methods used to elicit preferences for outcomes.	pg 14, lines 1-3
Estimating resources and costs	13a	*Single study-based economic evaluation:* Describe approaches used to estimate resource use associated with the alternative interventions. Describe primary or secondary research methods for valuing each resource item in terms of its unit cost. Describe any adjustments made to approximate to opportunity costs.	pg 14, lines 7-13
	13b	*Model-based economic evaluation:* Describe approaches and data sources used to estimate resource use associated with model health states. Describe primary or secondary research methods for valuing each resource item in terms of its unit cost. Describe any adjustments made to approximate to opportunity costs.	pg 14, lines 3-7
Currency, price date, and conversion	14	Report the dates of the estimated resource quantities and unit costs. Describe methods for adjusting estimated unit costs to the year of reported costs if necessary. Describe methods for converting costs into a common currency base and the exchange rate.	pg 14, lines 5-7
Choice of model	15	Describe and give reasons for the specific type of decision-analytical model used. Providing a figure to show model structure is strongly recommended.	pg 8, lines 18-19
Assumptions	16	Describe all structural or other assumptions underpinning the decision-analytical model.	pg 8-10
Analytical methods	17	Describe all analytical methods supporting the evaluation. This could include methods for dealing with skewed, missing, or censored data; extrapolation methods; methods for pooling data; approaches to validate or make adjustments (such as half cycle corrections) to a model; and methods for handling population heterogeneity and uncertainty.	pg 14 lines 15-24, pg 15 lines 1-2
**Results**			
Study parameters	18	Report the values, ranges, references, and, if used, probability distributions for all parameters. Report reasons or sources for distributions used to represent uncertainty where appropriate. Providing a table to show the input values is strongly recommended.	pg 21
Incremental costs and outcomes	19	For each intervention, report mean values for the main categories of estimated costs and outcomes of interest, as well as mean differences between the comparator groups. If applicable, report incremental cost-effectiveness ratios.	pg 15, lines 5-10
Characterising uncertainty	20a	*Single study-based economic evaluation:* Describe the effects of sampling uncertainty for the estimated incremental cost and incremental effectiveness parameters, together with the impact	pg 15, line 11-24, pg 16 lines 1-11

For peer review only

For peer review only - http://bmjopen.bmj.com/site/about/guidelines.xhtml

		of methodological assumptions (such as discount rate, study perspective).	
	20b	*Model-based economic evaluation:* Describe the effects on the results of uncertainty for all input parameters, and uncertainty related to the structure of the model and assumptions.	n/a
Characterising heterogeneity	21	If applicable, report differences in costs, outcomes, or cost-effectiveness that can be explained by variations between subgroups of patients with different baseline characteristics or other observed variability in effects that are not reducible by more information.	pg 15, lines 21-24, pg 16, lines 1-2
**Discussion**			
Study findings, limitations, generalisability, and current knowledge	22	Summarise key study findings and describe how they support the conclusions reached. Discuss limitations and the generalisability of the findings and how the findings fit with current knowledge.	pg 16, lines 13-19
**Other**			
Source of funding	23	Describe how the study was funded and the role of the funder in the identification, design, conduct, and reporting of the analysis. Describe other non-monetary sources of support.	pg 2, lines 2-13
Conflicts of interest	24	Describe any potential for conflict of interest of study contributors in accordance with journal policy. In the absence of a journal policy, we recommend authors comply with International Committee of Medical Journal Editors recommendations.	

For consistency, the CHEERS Statement checklist format is based on the format of the CONSORT statement checklist

The **ISPOR CHEERS Task Force Report** provides examples and further discussion of the 24-item CHEERS Checklist and the CHEERS Statement. It may be accessed via the *Value in Health* link or via the ISPOR Health Economic Evaluation Publication Guidelines – CHEERS: Good Reporting Practices webpage: http://www.ispor.org/TaskForces/EconomicPubGuidelines.asp

The citation for the CHEERS Task Force Report is:
Husereau D, Drummond M, Petrou S, et al. Consolidated health economic evaluation reporting standards (CHEERS)—Explanation and elaboration: A report of the ISPOR health economic evaluations publication guidelines good reporting practices task force. Value Health 2013;16:231-50.

For peer review only

BMJ Open

Chronic Hepatitis B virus case-finding in UK populations born abroad in intermediate or high endemicity countries: an economic evaluation

Journal:	*BMJ Open*
Manuscript ID	bmjopen-2019-030183.R1
Article Type:	Research
Date Submitted by the Author:	03-May-2019
Complete List of Authors:	Martin, Natasha; University of California San Diego; University of Bristol, Population Health Sciences<br>Vickerman, Peter; University of Bristol, Population Health Sciences<br>Khakoo, Salim; University of Southampton<br>Ghosh, Anjan; NHS London Borough of Bexley<br>Ramsay, Mary; Public Health England, immunisation; London School of Hygiene and Tropical Medicine, Epidemiology<br>Hickman, M; University of Bristol, Population Health Sciences<br>Williams, Jack; London School of Hygiene and Tropical Medicine<br>Miners, Alec; London School of Hygiene and Tropical Medicine
**Primary Subject Heading**:	Health economics
Secondary Subject Heading:	Health economics, Infectious diseases
Keywords:	Hepatology < INTERNAL MEDICINE, hepatitis b virus, HEALTH ECONOMICS, economic evaluation, case-finding, health services research

SCHOLARONE™
Manuscripts

Title: Chronic Hepatitis B virus case-finding in UK populations born abroad in intermediate or high endemicity countries: an economic evaluation

Authors: Natasha K Martin[1,2], Peter Vickerman[1], Salim Khakoo[3], Anjan Ghosh[4], Mary Ramsay[5], Matthew Hickman[2], Jack Williams[6] and Alec Miners[6]

Affiliations [1]Division of Infectious Diseases and Global Public Health, University of California San Diego, USA. [2]Population Health Sciences, University of Bristol, UK. [3]Clinical and Experimental Sciences, Faculty of Medicine, University of Southampton, Southampton, UK. [4]NHS London Borough of Bexley, London, UK. [5]Public Health England, London, UK. [6]Department of Health Services Research and Policy, London School of Hygiene and Tropical Medicine, London, UK.

Corresponding author: Dr Alec Miners, 15-17 Tavistock Place, Faculty of Public Health and Policy, London School of Hygiene & Tropical Medicine, London, WC1 9SH, UK

T: 00 44 (0) 0207 927 2069

E: Alec.miners@lshtm.ac.uk

Keywords: Hepatitis B virus, economic evaluation, case-finding and health services research

Abbreviations: HBV: Hepatitis B Virus; HBsAg: hepatitis B virus surface antigen; HBeAg: hepatitis B virus e antigen; ALT: alanine transaminase; DNA: deoxyribose nucleic acid

For peer review only

Financial Support: This work was originally funded by the UK National Institute for Health and Care Excellence. PV, MR and MH are affiliated with the National Institute for Health Research Health Protection Research Unit (NIHR HPRU) in Evaluation of Interventions at the University of Bristol in partnership with Public Health England (PHE). NM, PV, and MH acknowledge funding from National Institute for Drug Abuse R01 DA037773. NM also acknowledges funding from the University of California San Diego Center for AIDS Research(CFAR), a National Institute of Health (NIH) funded program [grant number P30 AI036214] , which is supported by the following NIH Institutes and Centers: NIAID, NCI, NIMH, NIDA, NICHD, NHLBI, NIA, NIGMS, and NIDDK. The views in this publication are those of the authors and not necessarily those of the NHS, the National Institute for Health Research, the Department of Health and Social Care or Public Health England. AM, PV and JW are members of the NIHR's Sexually Transmitted Infections and Blood Borne Virus Health Protection Research Unit.

Competing interests: NM and PV have received unrestricted research grants from Gilead, outside the submitted work. NM has received honoraria from Gilead and Merck. MH reports personal fees from Gilead, Abbvie, and MSD. AM, SK, AG, MR, JW have no disclosures.

Word Count: 3392 not including references.

Figures: 4

Tables: 1

For peer review only

ABSTRACT

Objectives: The majority (>90%) of new or undiagnosed cases of hepatitis B virus (HBV) in the United Kingdom (UK) are among individuals born in countries with intermediate or high prevalence levels (≥2%). We evaluate the cost-effectiveness of increased HBV case-finding among U.K. migrant populations, based on a one-time opt out case-finding approach in a primary care setting.

Design: Cost-effectiveness evaluation. A decision model based on a Markov approach was built to assess the progression of HBV infection with and without treatment as a result of case-finding. The model parameters, including the cost and effects of case-finding and treatment, were estimated from the literature. All costs were expressed in 2017/18 GBPs and health outcomes as quality-adjusted life-years (QALYs).

Intervention: HCV case-finding among U.K. migrant populations born in countries with intermediate or high prevalence levels (≥2%) in a primary care setting compared to no intervention (background testing).

Results: At a 2% hepatitis B surface antigen (HBsAg) prevalence, the case-finding intervention led to a mean incremental cost-effectiveness ratio (ICER) of £13,625 per QALY gained which was 87% and 98% likely of being cost-effective at willingness to pay (WTP) thresholds of £20,000 and £30,000 per additional QALY, respectively. Sensitivity analyses indicated that the intervention would remain cost-effective under a £20,000 WTP threshold as long as HBsAg prevalence among the migrant

For peer review only

population is at least 1%. However, the results were sensitive to a number of parameters, especially the time horizon and probability of treatment uptake.

Conclusions: HBV case-finding using a one-time opt out approach in primary care settings is very likely to be cost-effective among UK migrant populations with HBsAg prevalence ≥1% if the WTP for an additional QALY is around £20,000.

For peer review only

Strengths and limitations of this study

- Our cost-effectiveness evaluation is one of few studies evaluating HBV case-finding among populations born abroad in intermediate to high endemicity countries.
- Strengths include numerous sensitivity analyses assessing how cost-effectiveness varies for a range of different prevalences, intervention effect and cost, thus increasing the generalizability of our results to other similar interventions and different settings.
- A key limitations is uncertainty in the exact cost or effect of this intervention if scaled up to a national level.
- The model, due to a lack of available data, did not incorporate any additional impact of household contact tracing of diagnosed cases.
- The model also does not incorporate the possibility of simultaneous testing for hepatitis C virus.

INTRODUCTION

Worldwide, the burden of liver disease continues to rise and remains an urgent public health problem[1]. It is estimated that viral hepatitis is in the top 10 leading causes of mortality globally[2], the majority due to infection with hepatitis B virus (HBV)[3]. Chronic infection with HBV can lead to liver fibrosis, cirrhosis, hepatocellular carcinoma, and death in the absence of treatment. It is estimated that over 5% of the world's population are chronic carriers of HBV[4]. Globally, HBV burden is highest in low-middle income countries in areas such as Sub-Saharan African and East Asia[3]. HBV is spread through exposure to infected blood or body fluids, with the majority of chronic infections acquired perinatally or during childhood[1]. Recently, effective antiviral treatment for HBV has become available which may achieve long-term viral suppression and slow progression of disease[5 6].

The United Kingdom (UK) has a low burden of HBV, with an estimated 0.4% of adults infected with chronic hepatitis B (CHB)[7], and only approximately 320 cases of acute HBV reported in England in 2015[8]. The vast majority (80% to 90%) of newly diagnosed chronic HBV infections are among migrant individuals living in the UK that were born overseas in countries with intermediate (2-7%) or high HBV prevalence (≥8%) as defined by the World Health Organization[9], such as China or Pakistan[10-12]. Although uncertain, it is also likely that a considerable number of people with chronic HBV remain undiagnosed. For example, in one study in Bristol only 12% of migrants born in countries with endemic prevalence >2% had been tested for HBV[10]. Due to the often asymptomatic nature of chronic infection[13], individuals with HBV infection can often remain undiagnosed until they develop advanced liver disease. It is critical,

For peer review only

therefore, that increased case-finding among UK migrant populations is enhanced to ensure timely treatment and follow-up to prevent complications from liver disease.

The UK, like many countries worldwide, recommends universal screening of pregnant women to identify and immunize neonates exposed to HBV infection, which has been shown to be highly cost-effective and under some circumstances cost-saving[14]. However, the UK is one of only six countries in Europe which does not offer universal immunization against hepatitis B (along with Denmark, Finland, Iceland, Norway and Sweden). These countries have a very low HBV endemicity and so it is unlikely to be cost-effective to introduce a separate universal HBV vaccination programme[15]. Recent assessments of the cost-effectiveness of universal childhood HBV vaccination suggest that it may be cost-effective if introduced with other vaccines as a component of a hexavalent vaccine – the UK moved to such a product in 2017[15]. Nonetheless, infant vaccination is unlikely to have a great impact on the prevalence of chronic HBV in countries such as the UK because few transmissions are thought to occur once people have entered the country[16]. For these reasons, there remains a critically important role for case-finding activities.

While studies in The Netherlands have shown the cost-effectiveness of one-time screening programs (where a test offer is mailed to migrant individuals identified through a population registry[17]), until recently there has not been a published evaluation from a UK perspective. This changed earlier this year when the results of a randomized controlled trial (HepFREE) showed that incentivized screening of HBV and HCV in first and second-generation migrants in a primary care setting was shown to be effective and cost-effective in the UK; the incentive included a startup

payment of £500 per general practice, £25 for each enrolled participant and support from a dedicated clinician 3 days a week[18]. However, in contrast to an incentivized screening approach, pilot data from the UK also indicates that an opt-out HBV case-finding approach in primary care settings without incentives was also highly effective, and potentially a less expensive approach[19]. Additionally, it was unclear in the previous analysis for the HepFREE trial how much the cost-effectiveness was driven by HCV versus HBV outcomes, and whether the intervention was cost-effective for HBV alone. Further, it is unknown how the cost-effectiveness of HBV case-finding could vary for a range of prevalences (which likely vary by country of origin), costs, and uptake rates that may occur when the interventions are rolled out across different settings.

The aim of this paper is to evaluate the cost-effectiveness of increased HBV case-finding among UK migrant populations born in intermediate or high endemicity countries, based on a one-time opt out case-finding approach in primary care settings. Importantly, to increase the generalizability of our results to other similar interventions and different settings, we assess how the cost-effectiveness of HBV case-finding varies for a range of different prevalences, intervention effect and cost.

METHODS

The economic evaluation was undertaken using a Markov approach, where a closed cohort of U.K. individuals born in countries with intermediate or high prevalence levels (≥2%) move between a set of discrete health states representing HBV infection stage[20 21]. A UK National Health Service's cost perspective was used. All costs were displayed in GBP 2017/18 prices and a 40-year time horizon was used

For peer review only - http://bmjopen.bmj.com/site/about/guidelines.xhtml

with an annual time step. Health outcomes were expressed in terms of Quality-Adjusted Life-Years (QALYs). QALYs and costs were discounted at 3.5% per annum according to UK National Institute for Health and Care Excellence (NICE) recommendations[22]. Uncertainty in the results was examined using deterministic and probabilistic sensitivity analysis (PSA); distributions shown in the tables relate to the PSA analysis. Each PSA consisted of 5,000 runs. HBV transmission was not included in the model as most infections are likely to occur in UK migrant populations before entering the U.K.[16]. Ethical approval was not required for this study as it is an economic modelling exercise utilising published evidence and aggregate data from Public Health England.

Intervention and target population

A systematic literature review found few studies evaluating HBV case-finding in migrant or other high-risk populations, nor have many studies been published since this review[18 23]. Our study evaluates the cost-effectiveness of HBV case-finding in the U.K. for individuals born in countries with intermediate or high prevalence levels (≥2%). The base case analysis uses the results from an uncontrolled study in which Pakistani/British Pakistani people registered at general practices (GPs) in London's East End were written to and invited to 'opt out' of being tested for hepatitis B and C infection. Those who did not opt out were telephoned and asked to attend a clinic for testing[19]. The intervention was designed to increase the likelihood of testing for each infection, assumed in this analysis to occur over the initial model cycle of one year. After this time, the intervention effect was assumed to be zero, with the probability of testing reverting to background levels. The comparator programme or 'no intervention' was defined as the background likelihood of testing through existing

routes such as sexual health or genitourinary medicine clinics, antenatal clinics or primary care[24]. Although we base our analysis on data from a study among Pakistani/British Pakistani individuals in London, we evaluate the potential impact of this intervention in populations with a range of HBV prevalences as observed among UK migrants born in countries with intermediate or high prevalence levels (>=2%).

Model structure

The Markov model was created to represent HBV disease progression and current understanding of policies regarding disease management (Figure 1). The natural history element of the model was largely based on a model developed by Shepherd et al.[25 26] The model simulates a cohort of people, a proportion of whom are positive for hepatitis B surface antigen (HBsAg+). For this analyses, we refer to "HBV prevalence" as the proportion of individuals who are HBsAg+. Individuals who are negative for hepatitis B surface antigen.(HBsAg-) remain in the model with a general population level of mortality but incurring no HBV-related costs, other than the possibility of being tested for infection. Known HBsAg+ people were assumed to undergo a full viral profile when initially diagnosed. Acute HBV infection was not included in the model as it is likely that people would have been infected much longer than 6 months ago.

Among HBsAg+ individuals, the model stratifies by utually exclusive stages of chronic hepatitis B (CHB), including: HBeAg seroconverted (where ALT levels and HBV DNA are both low), active CHB hepatitis B e-antigen positive (HBeAg+) disease, active CHB hepatitis B e-antigen negative (HBeAg-) disease, and inactive CHB HBeAg- (where ALT levels and HBV DNA are both low). Individuals progressed

For peer review only

from CHB to compensated cirrhosis, decompensated cirrhosis (DC), hepatocellular carcinoma (HCC), liver transplant, and post-transplant stages if appropriate drug treatment was not initiated or failed. Due to the severity of the disease and likely presentation, the infection status of all individuals with CHB was assumed to become known when they developed DC, HCC or required a liver transplant. Individuals could die from non-HBV related causes from any health state.

Individuals who had raised ALT and HBV (active) levels and who were CHB HBeAg+ were assessed for fibrosis and offered treatment with pegylated interferon for the first year, followed by tenofovir until seroconversion is achieved (as per NICE guidelines[27]) or later stage CHB developed. We assumed successful treatment of these individuals resulted in normalization of ALT and lowering of HBV DNA levels, therefore resulting in transition to the HBeAg seroconverted stage. Individuals with no evidence of compensated cirrhosis stopped treatment at this time[27]. Individuals with active CHB who were HBeAg- also received pegylated interferon for the first year, followed by tenofovir if they had not developed inactive CHB HBeAg- disease[27]. However, even following the development of inactive disease, they were assumed to stay on treatment indefinitely to sustain the achieved level of viral suppression[27]. Individuals with evidence of compensated cirrhosis were assumed to remain on tenofovir as long as no further disease progression was recorded, irrespective of e-antigen status[27]. All individuals were assumed to stop treatment on progression to DC or later stages of disease.

Individuals with CHB whose infection status was unknown and those that tested HBsAg+, but declined treatment, were assumed to develop progressive disease

For peer review only - http://bmjopen.bmj.com/site/about/guidelines.xhtml

according to a set of defined transition probabilities, with different probabilities used for those who accepted treatment. (Supplementary Tables 1 and 2). As the focus of this analysis is on case-finding, we do not model possible adverse events associated with treatment or treatment resistance.

Model parameters

HBV prevalence among migrant populations to the UK

There is substantial heterogeneity in HBV burden between different migrant populations in the UK depending on their country of origin. Additionally, HBV prevalence among UK migrants may be different compared with their country of origin; a recent UK study of antenatal testing showed the prevalence in migrants was generally less than published estimates for the country of origin, with only Eastern Asia having a higher than expected prevalence[11]. Public Health England (PHE) data on those undergoing routine diagnostic testing suggests that the HBV prevalence among all Asian or British Asian people in the UK is approximately 2%, however these data do not specify country of origin in any further detail[28]. By contrast, the HBV prevalence estimates obtained through targeted studies or antenatal testing have identified a range of prevalence among UK migrants born in countries with intermediate-high HBV endemicity, such as 17% (Vietnam-born), 7%-10%[29 30] (China-born), 3-6% (Somalia-born), 1-3% (Pakistan-born), 0.5-1.5% (Bangladesh-born), 0.7% (Poland-born), and 0.5% (India-born)[16 31-33]. The recent HepFREE trial found a lower prevalence of 1.1%, varying by country of origin, although this included second generation migrants that were born in the UK[18].

For peer review only - http://bmjopen.bmj.com/site/about/guidelines.xhtml

Due to the uncertainty in prevalence within populations, and the likely wide variation between populations, in the base case, we assume an HBV prevalence (HBsAg+) of 2%, but explore a range of values (from 0.05-10%) in the sensitivity analysis.

Transition probabilities

Transition probability values, representing the likelihood of moving between health states, for untreated disease stages were based on those reported in a 2006 UK Health Technology Assessment report (Supplementary Tables 1 and 2)[25].

Background testing rate and diagnostic accuracy

The background rate of testing for migrants in the absence of the intervention was estimated using data from PHE, indicating a probability of 2.6% per year[24]. The HBsAg diagnostic test was assumed to be 100% accurate.

Referral and treatment effect

Few studies have quantified the number of people diagnosed with CHB who are subsequently referred to, and accept, appropriate further clinical investigations for their infection. However, interruptions in the cascade of care post-diagnosis are known to be an issue in the management of CHB and hepatitis C virus (HCV) infection both in the U.K. and elsewhere, particularly in migrant populations[34]. We therefore include a single probability of being referred for specialist care following a HBsAg+ test result, attending the appointment, and starting treatment for those eligible. In the absence of HBV related data, the we utilize data on the proportion of individuals who ho were identified using algorithmic approaches as being Asian and who tested positive and subsequently received treatment for chronic HCV from

For peer review only

2004-2015 (0.42, based on data supplied by Public Health England, personal communication with Public Health England staff). However, we consider this parameter to be highly uncertain and undertake sensitivity analysis around it using a wide range of alternative values (10% to 60%).

While a systematic review and meta-analysis of the effects of drug therapy for CHB is available[35], we estimated the impact of antiviral treatment using data from a study which a much longer follow up period (5 years rather than 1 year)[36]. For HBeAg+ individuals, we assumed 20% would e-antigen seroconvert after 1-year of treatment with pegylated interferon and 5.4%/year following treatment with tenofovir, resulting in 40% having seroconverted by 5 years. For HBeAg- individuals, we assumed a 75% probability of response (development of inactive disease) following the initial 1-year of pegylated interferon and 2.3%/year following treatment with tenofovir Therefore, we assumed that 84% would develop inactive disease by 5 years. Irrespective of whether individuals were HBeAg+ or HBeAg-, they were assumed to continue treatment after year 5 with tenofovir until they responded to it assuming the same constant rate of response.

The probability of responding to treatment was assumed to be the same for people with or without compensated disease. However, once people developed compensated disease, it was assumed not to regress following treatment, and the costs and disutility associated with it would remain. The only benefit of treatment in this group was slower progression to poorer health states compared with not being treated.

For peer review only

Intervention effect

The base case probability of testing for HBsAg in the intervention arm was based on a one-time 'opt out' option within a general practice setting; 223 out of 1,134 (19.7%) eligible tested after being identified using a GP registries database and responding to a written invite[19].

Cohort demographics and initial stage distribution

PHE data suggests that the average age at HBV diagnosis in the UK Asian population is approximately 35 years of age[28], which we use as the base-case starting age in our model but vary in the sensitivity analysis. The proportion of people with CHB who were HBeAg+ in our starting cohort was assumed to be 0.14 ([71/490] personal communication with Public Health England staff). The proportion of people who had seroconverted, or developed inactive disease, before being tested for HBsAg, was assumed to be 80% (personal communication with Public Health England staff). It was further assumed that 44% of people with active HBeAg+ or HBeAg- disease, had already developed compensated cirrhosis[37].

Health utilities and costs

Utility values related to HBV infection were sourced from the review by Shepherd et al[25] and Takeda et al[26] (Supplementary Table 3). The costs of HBV testing/monitoring, antiviral treatment, and health-state specific costs were taken from a number of published sources[25 37] (Table 1), inflated to GBP £2017 where appropriate using the NHS Hospital and Community Health Services Pay and Prices Index and the Health Service Cost Index[38 39]. The intervention cost was estimated at £4 per person eligible for testing. This cost relates to the resources required to

For peer review only

identify and invite each individual for a test and excludes the cost of any tests and treatments. Thus, if 100 individuals were eligible for testing, the total cost of the intervention was £400 irrespective of how many people attended for a test. The importance of this assumption was assessed in the sensitivity analysis given the extent of uncertainty.

Main outcomes

Our main results incorporate a probabilistic sensitivity analysis (PSA), in which relevant parameters are simultaneously sampled 5,000 times to represent underlying uncertainty, including the costs, utilities probabilities and disease progression parameters. We present total and incremental costs, QALYs, and incremental cost-effectiveness ratios (ICERs). Mean and 2.5-97.5% centile (95% CI) results are presented. We additionally present the proportion of simulations which are cost-effective under £20,000 and £30,000 WTP thresholds.

Sensitivity analyses

To test the robustness of the results to alternative assumptions, we undertook extensive one-way sensitivity analyses on starting age, discount rate, drug cost, time horizon, treatment uptake, intervention effect, and intervention cost. Finally, due to the uncertainty surrounding the intervention cost and impact if scaled-up to the national level and among different migrant populations, we undertook a threshold analysis where we evaluated the minimum HBV prevalence at which the intervention remains cost-effective at a willingness to pay (WTP) threshold of <£20,000 per QALY gained with varying intervention cost (between £1 and £20, £4 per person eligible at base-case), intervention effect (between 5% and 30%, 19.7% uptake at base-case)

For peer review only

and HBsAg prevalence (between 1% and 10%, 2% base-case). We displayed the results of this sensitivity analysis as a contour map.

RESULTS

Base-case 2% HBsAg prevalence

At a 2% HBsAg prevalence, the HBV case-finding intervention resulted in mean incremental costs and QALYs of about £28 and 0.002 respectively over the 5,000 samples, corresponding to an ICER of £13,625 per QALY gained (95%CI £7,121-27,588). The intervention was 87% and 98% likely to be cost-effective at £20,000 and £30,000 WTP per additional QALY thresholds, respectively (Supplementary Figure 1). Most of the univariate sensitivity analyses produced ICERs below a £20,000 WTP threshold (Figure 2), including reducing the likelihood of testing from 19.7% to 5% (£19,323/QALY gained). However, the exceptions were assuming a 20-year time horizon instead of 40 years (£22,713/QALY gained), discounting QALYs at 6% instead of 3.5% (£21,970/QALY gained), not discounting costs instead of 6% (21,521/QALY gained) and doubling the costs of all drug treatments from £3,979/£2,453 to £7,957/ £4,905 (£22,586/QALY gained). Decreasing the probability of treatment uptake after testing positive for HBsAg from 0.42 to 0.1 increased the ICER to over £30,000 (£31,340/QALY gained).

Impact of variation in HBV prevalence and intervention impact (cost, effect and uptake)

Cost-effectiveness of HBV case-finding was strongly driven by HBV prevalence. Our sensitivity analyses indicated that the intervention would remain cost-effective under

For peer review only

a £20,000 WTP threshold as long as HBV prevalence among the migrant population is equal to or exceeds 1% (Figure 3).

Due to the uncertainty in cost and intervention impact if scaled-up across the U.K. and among different migrant population, we additionally present a sensitivity analysis of the threshold HBV prevalence which would ensure the intervention is cost-effective under a £20,000 WTP with varying costs and intervention effects (Figure 4). The contour map shows that, for example, the intervention would be cost-effective at a prevalence of 1% if it cost £6 per person and the intervention effect was 20%. However, it would no longer be cost-effective at a 1% prevalence level and £6 cost if the intervention effect reduced to 10%.

DISCUSSION

HBV case-finding using a one-time opt out approach in primary care settings has a high potential to be cost-effective among U.K. migrant populations with a HBV prevalence at or above an average of 1%. However, the results are sensitive to a number of factors including the intervention effect or cost, rate of treatment uptake, assuming a much shorter time horizon and (unrealistically) high discount rates and drug costs.

Limitations

The main limitation with the analysis is the substantial uncertainty surrounding the costs of the intervention and its effect if this case-finding intervention were scaled-up to a national level. Nonetheless, extensive sensitivity analysis shows that the intervention remained cost-effective across a large range of evaluated scenarios.

For peer review only - http://bmjopen.bmj.com/site/about/guidelines.xhtml

Thus, while establishing more robust estimates of the costs and effects of interventions to find cases of HBV will undoubtedly decrease the uncertainty around our results, we believe the scope for the modelled intervention to be cost-effective is extremely high.

Current U.K. NHS HBV-testing policy is to contact household members once a case has been identified. However, we were unable to include this aspect in our analysis due to a lack of data specific to the target migrant populations on the size and age distribution of households of infected contacts, the probability that contacts were HBsAg+ and the likelihood that contacts could be traced in the first instance. The impact of excluding this process on the ICER we report is difficult to determine. For example, if contact tracing results in a high proportion of people being treated for CHB the ICER could decrease. Conversely, if many HBsAg- people are vaccinated against HBV, the ICER could increase as there is already evidence to suggest it is unlikely to be cost-effective[15].

Finally, we did not model the possibility of simultaneously testing for hepatitis C virus (HCV), which may increase the cost-effectiveness of the intervention though evidence on the HCV prevalence among migrants also has uncertainies[19].

Comparison with other studies

Five studies have examined the cost-effectiveness of screening for HBV among migrant populations. A Dutch study[17] found that screening migrants from countries with high or intermediate HBV prevalence (assuming a 3.4% chronic infection prevalence) was highly cost-effective (EUR9000 per QALY gained) at a screening

For peer review only

campaign cost of approximately EUR11 per person eligible and 35% uptake – which is consistent with our sensitivity analysis. Another study explored screening and treatment of migrants from Asian and Pacific Islands in the US[40], finding it to be cost-effective (US$36,000 per QALY gained) but also assuming a much higher prevalence of HBV (10%), screening uptake (70%) and no screening programme costs aside from the diagnostic tests. Two studies examined the cost-effectiveness of screening all migrants to Canada[41 42], both finding tenofovir-based treatment moderately cost-effective (CAD$40,000/QALY [~£22,000]) at 4.8-6.5% chronic infection prevalence's. Our model assumes a lower prevalence of chronic HBV, higher treatment efficacy and lower treatment and screening costs than the North American studies, which may explain the difference in cost-effectiveness estimates. Lastly, our results are partially consistent with findings from the recent HepFREE trial, which was found to be cost-effective (£8,540/QALY) for a similar observed intervention effect (19.7% uptake of testing compared to 19.5% uptake in our study). However, HepFREE had higher intervention costs (>£25 per patient compared to £1-20 in our model), combined HCV and HBV screening and identified patients on basis of ethnic group rather than country of birth[43].

Conclusions

Our analysis suggests that interventions to increase HBV case-finding in primary care among UK migrant populations with a prevalence of at least 1% – such as using a one-time opt out approach – could be cost-effective, underpinning current National Institute for Health and Care Excellence guidance[44]. Critically, at a threshold prevalence above 1% this will encompass migrant populations from most countries with endemic HBV, even if there is a healthy migrant effect (with migrant populations

For peer review only

in UK on average at lower risk than people in their country of origin[16]). These recent results support the recommendation that interventions to increase HBV case-finding in primary care among U.K. migrant populations should be expanded, but needs to be based on screening by country of birth rather than ethnic group.

For peer review only

Author contributions: AM, PV, MH designed the study. AM, AG, JW and NM coded the analysis. All authors (AM, PV, MH, AG, JW, SK, MR, NM) interpreted the data. AM and NM wrote the first draft. All authors (AM, PV, MH, AG, JW, SK, MR, NM) contributed to the manuscript drafting, approved of the final version, and agreed to authorship.

Data Sharing: Model code available on request to the corresponding author.

License statement: I, the Submitting Author has the right to grant and does grant on behalf of all authors of the Work (as defined in the below author licence), an exclusive licence and/or a non-exclusive licence for contributions from authors who are: i) UK Crown employees; ii) where BMJ has agreed a CC-BY licence shall apply, and/or iii) in accordance with the terms applicable for US Federal Government officers or employees acting as part of their official duties; on a worldwide, perpetual, irrevocable, royalty-free basis to BMJ Publishing Group Ltd ("BMJ") its licensees and where the relevant Journal is co-owned by BMJ to the co-owners of the Journal, to publish the Work in BMJ Open and any other BMJ products and to exploit all rights, as set out in our licence.

The Submitting Author accepts and understands that any supply made under these terms is made by BMJ to the Submitting Author unless you are acting as an employee on behalf of your employer or a postgraduate student of an affiliated institution which is paying any applicable article publishing charge ("APC") for Open Access articles. Where the Submitting Author wishes to make the Work available on

For peer review only

an Open Access basis (and intends to pay the relevant APC), the terms of reuse of such Open Access shall be governed by a Creative Commons licence – details of these licences and which Creative Commonslicence will apply to this Work are set out in our licence referred to above.

Patient and Public Involvement: This study was commissioned by the UK National Institute for Health and Care Excellence (https://www.nice.org.uk/guidance/ph43/) with representation from lay members on the guidance panel who contributed to shaping the proposed intervention and interpretation of the study findings and implications.

For peer review only

1

For peer review only

FIGURE LEGENDS

Figure 1. HBV model schematic. The arrows denote possible transitions between states; HBsAg, hepatitis B virus surface antigen; HBeAg: hepatitis b virus e antigen; CHB, chronic hepatitis B virus; CC, compensated cirrhosis; DC, decompensated cirrhosis; HCC, hepatocellular carcinoma; LT, liver transplant; *individuals may or may not know their infection status; %individuals with CC responding to treatment were assumed to keep the costs and utility associated with CC, but with disease progression probabilities equivalent to HBeAg seroconversion / inactive disease; “transitions permitted from all health states to death

Figure 2: Univariate sensitivity analysis on the ICER with a 2% HBV prevalence scenario. ICER, incremental cost-effectiveness ratio; Y-axis indicates the base case ICER of £21,400 per QALY gained; *halves or doubles all baseline drug costs where relevant

Figure 3. Mean incremental cost-effectiveness ratio (ICER) of HBV screening by varying HBsAg prevalence

Figure 4. Contour map showing for a range of costs (horizontal axis) and intervention effects (vertical axis), the threshold HBV prevalence (contours) where the intervention ICER falls under a £20,000 willingness to pay threshold.

For peer review only

Table 1: Annual costs in 2017/18 UK prices (£)

Cost	Mean	95% interval of sampled range^	Source
**Intervention cost per person eligible for testing***	4	-	Assumption
**HBsAg test (laboratory)**	10	-	Assumption
**Pegylated interferon**	3,979	-	BNF[45]
**Tenofovir**	2,453	-	BNF[45]
**ALT and ultrasound**	77	-	Assumption[45]
**Full viral profile**	432	-	Assumption[45]
**HBeAg+ seroconverted / HBeAg- ALT/DNA low[a]**	335	240-446	Shepherd[25]
**HBeAg+ / HBeAg- active disease[b]**	674	480-896	Shepherd[25]
**Compensated cirrhosis**	1,606	1,052-2,283	Crossan[37]
**Decompensated cirrhosis**	38,212	21,848-60,645	Crossan[37]
**Hepatocellular carcinoma**	38,212	21,848-60,645	Crossan[37]
**Liver transplant (first year)**	67,698	57,301-79,287	Crossan[37]
**Liver transplant (subsequent years)**	17,231	5,415-35,399	Crossan[37]

*Indicates a one off cost; ^Sampled values from the probabilistic sensitivity analysis using a gamma distribution; [b]costs are additional to[a]

For peer review only

References

1. World Health Organisation. Global hepatitis report, 2017 [Available from: http://apps.who.int/iris/bitstream/10665/255016/1/9789241565455-eng.pdf?ua=1 accessed 04/08/2017].
2. Cooke GS, Lemoine M, Thursz M, et al. Viral hepatitis and the Global Burden of Disease: a need to regroup. *J Viral Hepat* 2013;20(9):600-1. doi: 10.1111/jvh.12123
3. Lozano R, Naghavi M, Foreman K, et al. Global and regional mortality from 235 causes of death for 20 age groups in 1990 and 2010: a systematic analysis for the Global Burden of Disease Study 2010. *The Lancet* 2012;380(9859):2095-128. doi: 10.1016/S0140-6736(12)61728-0
4. World Health Organisation. Hepatitis B fact sheet 2014 [Available from: http://wwwwhoint/mediacentre/factsheets/fs204/en/2014 accessed 29/04/2019].
5. Rajbhandari R, Chung RT. Treatment of Hepatitis B: A Concise Review. *Clin Transl Gastroenterol* 2016;7(9):e190. doi: 10.1038/ctg.2016.46
6. World health Organisation. Guidelines for the prevention, care and treatment of persons with chronic hepatitis B infection. Geneva. 2015 [Available from: http://apps.who.int/iris/bitstream/10665/154590/1/9789241549059_eng.pdf?ua=1&ua=1 accessed 16/06/2017].
7. Health Protection Agency. Migrant Health: Infectious diseases in non-UK born populations in the UK. An update to the baseline report 2011 [Available from: https://webarchive.nationalarchives.gov.uk/20140714091407/http://www.hpa.org.uk/webw/HPAweb&HPAwebStandard/HPAweb_C/1317131996733 accessed 24/09/2019].
8. Public Health England. Acute hepatitis B (England): annual report 2017 [Available from: https://assets.publishing.service.gov.uk/government/uploads/system/uploads/attachment_data/file/736145/hpr3118_hepB.pdf accessed 29/04/2019].
9. World Health Organisation. Heptatits B. Geneva. 2002 [Available from: https://apps.who.int/iris/bitstream/handle/10665/67746/WHO_CDS_CSR_LYO_2002.2_HEPATITIS_B.pdf;jsessionid=F89A0070DDEE2B34F204EC538ECD248D?sequence=1#page=1&zoom=auto,-149,603 accessed 29/04/2019].
10. Evlampidou I, Hickman M, Irish C, et al. Low hepatitis B testing among migrants: a cross-sectional study in a UK city. *Br J Gen Pract* 2016 doi: 10.3399/bjgp16X684817
11. Cochrane A, Evlampidou I, Irish C, et al. Hepatitis B infection prevalence by country of birth in migrant populations in a large UK city. *J Clin Virol*. 2015;68:79-82.
12. Hahne S, Ramsay M, Balogun K, et al. Incidence and routes of transmission of hepatitis B virus in England and Wales, 1995-2000: implications for immunisation policy. *J Clin Virol*. 2004;29:211-20.
13. Fattovich G. Natural history of hepatitis B. *J Hepatol* 2003;39:50-58. doi: 10.1016/S0168-8278(03)00139-9
14. Edmunds WJ, Ramsay R. The estimated cost-effectiveness of vaccination in infants born to hepatitis B positive mothers 2009 [Available from:

For peer review only

https://www.nice.org.uk/guidance/ph21/evidence/economic-analysis-3-full-report-371340255 accessed 29/04/2019].
15. Siddiqui MR, Gay N, Edmunds WJ, et al. Economic evaluation of infant and adolescent hepatitis B vaccination in the UK. *Vaccine* 2011;29(3):466-75.
16. Uddin G, Shoeb D, Solaiman S, et al. Prevalence of chronic viral hepatitis in people of south Asian ethnicity living in England: the prevalence cannot necessarily be predicted from the prevalence in the country of origin. *J Viral Hepat* 2010;17:327-35.
17. Veldhuijzen IK, Toy M, Hahné SJM, et al. Screening and Early Treatment of Migrants for Chronic Hepatitis B Virus Infection Is Cost-Effective. *Gastroenterology* 2010;138(2):522-30. doi: 10.1053/j.gastro.2009.10.039
18. Flanagan S, Kunkel J, Appleby V, et al. Case finding and therapy for chronic viral hepatitis in primary care (HepFREE): a cluster-randomised controlled trial. *Lancet Gastroenterol Hepatol*. 2019;4(1):32-44. doi: 10.1016/s2468-1253(18)30318-2
19. Lewis H, Burke K, Begum S, et al. What is the best method of case finding for chronic viral hepatitis in migrant communities? *Gut* 2011;Vol 60 Suppl 2:A26.
20. Beck JR, Pauker SG. The Markov process in medical prognosis. *Med Decis Making* 1983;3(4):419-58.
21. Sonnenberg FA, Beck JR. Markov models in medical decision making: a practical guide. *Med Decis Making* 1993;13(4):322-38.
22. National Institute for Health and Care Excellence. Guide to the methods of technology appraisal 2013 [Available from: https://www.nice.org.uk/process/pmg9/chapter/foreword accessed 29/04/2019].
23. Jones L, Bates G, McCoy E, et al. A systematic review of the effectiveness & cost-effectiveness of interventions aimed at raising awareness and engaging with groups who are at an increased risk of hepatitis B and C infection: Liverpool John Moores University, Centre for Public Health; 2011 [Available from: https://www.nice.org.uk/guidance/ph43/evidence/evidence-review-2-69062510 accessed 23/03/2017].
24. Health Protection Agency. Sentinel Surveillance of Hepatitis Testing in England - Hepatitis B and D 2010 Report - Analysis of testing between 2007 and 2010 2011 [Available from: https://webarchive.nationalarchives.gov.uk/20140714073050/http://www.hpa.org.uk/webc/HPAwebFile/HPAweb_C/1313155292332 accessed 29/04/2019].
25. Shepherd J, Jones J, Takeda A, et al. Adefovir dipivoxil and pegylated interferon alfa-2a for the treatment of chronic hepatitis B: a systematic review and economic evaluation. *Health Technol Assess* 2006;10(28)
26. Takeda A, Jones J, Shepherd J, et al. A systematic review and economic evaluation of adefovir dipivoxil and pegylated interferon-alpha-2a for the treatment of chronic hepatitis B. *J Viral Hepat* 2007;14:75-88.
27. National Institute for Health and Care Excellence. Hepatitis B (chronic): diagnosis and management 2013 [Available from: https://www.nice.org.uk/guidance/Cg165 accessed 24/03/2017].

For peer review only

28. Public Health England. Annual report from the sentinel surveillance study of blood borne virus testing in England: data for January to December 2015 2016 [Available from: https://www.gov.uk/government/uploads/system/uploads/attachment_data/file/540332/hpr2416_bbvs.pdf accessed 02/06/2017].
29. McPherson S, Valappil M, Moses S, et al. CHASE-B (Chinese hepatitis awareness, surveillance, and education): A pilot of targeted case finding for hepatitis B virus (HBV) in the British-Chinese community. *Gut* 2010;60:A25-A26.
30. Kawsar MT, Goh BT. Hepatitis B virus infection among Chinese residents in the United Kingdom. *Sex Transm Infect* 2002;78:166-68.
31. Aweis D, Brabin BJ, Beeching NJ, et al. Hepatitis B prevalence and risk factors for HBsAg carriage amongst Somali households in Liverpool. *Commun Dis Public Health*. 2001;4(4):247-52.
32. Brabin B, Beeching NJ, Bunn JE, et al. Hepatitis B prevalence among Somali households in Liverpool. *Arch Dis Child*. 2002;86(1):67-8.
33. McPherson S, Valappil M, Moses SE, et al. Targeted case finding for hepatitis B using dry blood spot testing in the British-Chinese and South Asian populations of the North-East of England. *J Viral Hepat* 2013;20(9):638-44. doi: 10.1111/jvh.12084
34. Vedio A, Liu EZH, Lee ACK, et al. Improving access to health care for chronic hepatitis B among migrant Chinese populations: A systematic mixed methods review of barriers and enablers. *J Viral Hepat* 2017;24(7):526-40. doi: 10.1111/jvh.12673
35. Woo G, Tomlinson G, Nishikawa Y, et al. Tenofovir and entecavir are the most effective antiviral agents for chronic hepatitis B: a systematic review and Bayesian meta-analyses. *Gastroenterology* 2010;139(4):1218-29.
36. Marcellin P, Gane E, Buti M, et al. Regression of cirrhosis during treatment with tenofovir disoproxil fumarate for chronic hepatitis B: a 5-year open-label follow-up study. *Lancet* 2013;381(9865):468-75. doi: 10.1016/s0140-6736(12)61425-1
37. Crossan C, Tsochatzis EA, Longworth L, et al. Cost-effectiveness of non-invasive methods for assessment and monitoring of liver fibrosis and cirrhosis in patients with chronic liver disease: systematic review and economic evaluation. *Health Technol Assess* 2015;19(9) doi: 10.3310/hta19090
38. Curtis L, Burns A. Unit costs of health and social care 2015 [Available from: http://www.pssru.ac.uk/project-pages/unit-costs/2015/ accessed 27/03/2017].
39. Curtis L, Burns A. Unit costs of health and social care 2018 [Available from: https://www.pssru.ac.uk/pub/uc/uc2018/sources-of-information.pdf accessed 18/12/2018].
40. Hutton DW, Tan D, So SK, et al. Cost-effectiveness of screening and vaccinating Asian and Pacific Islander adults for hepatitis B. *Ann Intern Med* 2007;147(7):460-9.
41. Rossi C, Schwartzman K, Oxlade O, et al. Hepatitis B screening and vaccination strategies for newly arrived adult Canadian immigrants and refugees: a cost-effectiveness analysis. *PLoS One* 2013;8(10) doi: 10.1371/journal.pone.0078548

For peer review only

42. Wong WWL, Woo G, Jenny Heathcote E, et al. Cost effectiveness of screening immigrants for hepatitis B. *Liver Int* 2011;31(8):1179-90. doi: 10.1111/j.1478-3231.2011.02559.x
43. Hickman M, Mandel S, Vickerman P, et al. Hepatitis case finding among migrants in primary care. *Lancet Gastroenterol Hepatol*. 2019;4(1):3-4. doi: 10.1016/s2468-1253(18)30385-6
44. National Institute for Health and Clinical Excellence. Hepatitis B and C - ways to promote and offer testing 2012 [Available from: http://publications.nice.org.uk/hepatitis-b-and-c-ways-to-promote-and-offer-testing-to-people-at-increased-risk-of-infection-ph43].
45. Royal Pharmaceutical Society. British National Formulary no. 71 2016 [Available from: www.bnf.org accessed 01/08/2016].

For peer review only

For peer review only

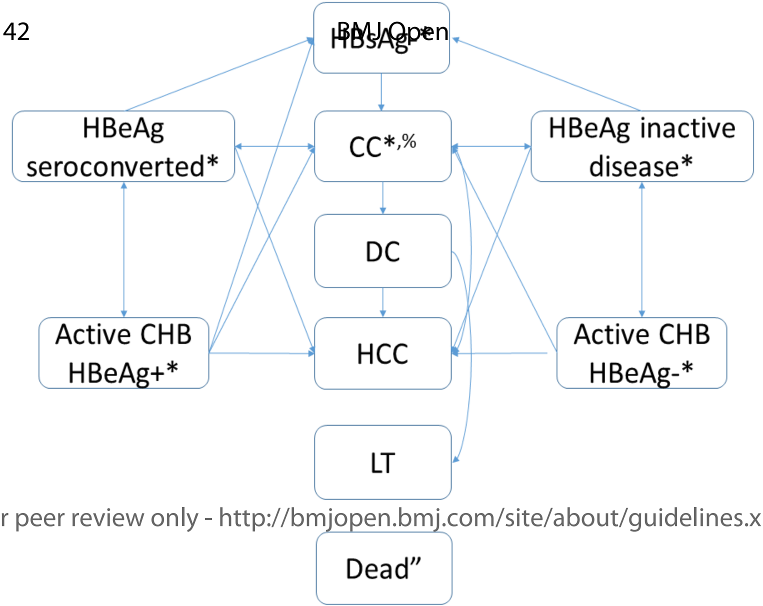

For peer review only - http://bmjopen.bmj.com/site/about/guidelines.xhtml

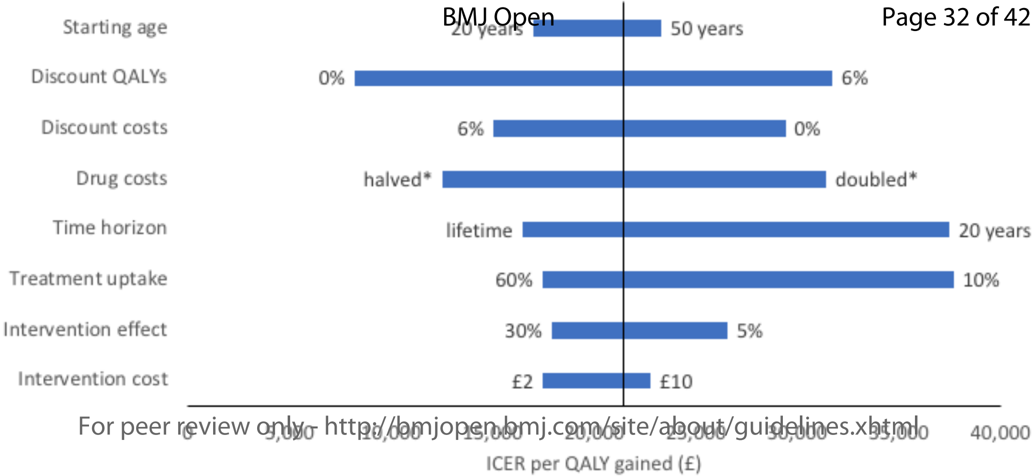

For peer review only - http://bmjopen.bmj.com/site/about/guidelines.xhtml

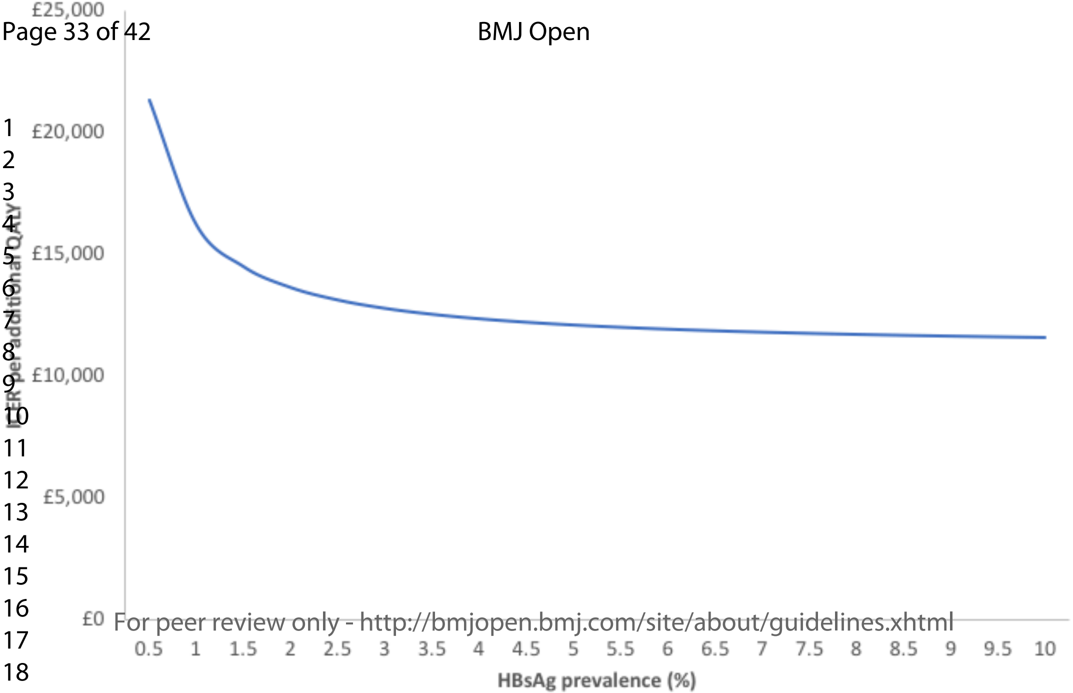
£25,000
£20,000
£15,000
£10,000
£5,000
£0
0.5 1 1.5 2 2.5 3 3.5 4 4.5 5 5.5 6 6.5 7 7.5 8 8.5 9 9.5 10
HBsAg prevalence (%)

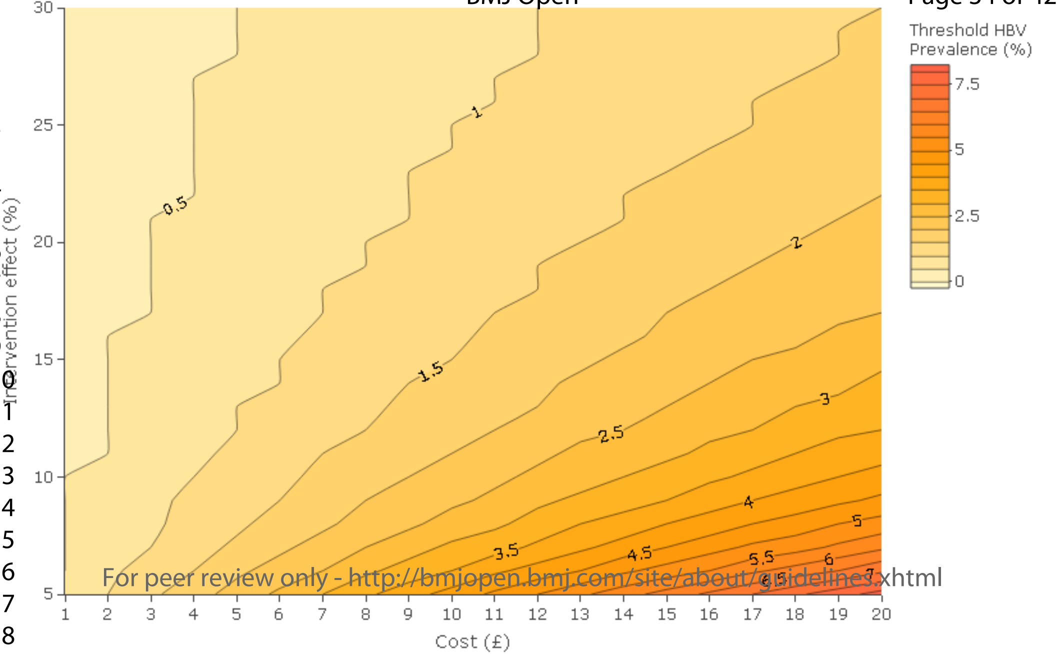

For peer review only - http://bmjopen.bmj.com/site/about/guidelines.xhtml

Supplementary Materials

Supplementary Table 1: Annual transition probability matrix for people who enter the model as HBsAg+ and HBeAg+ derived from Shepherd(1) and Marcellin(2)

To: / From:	HBsAg seroconverted	HBeAg seroconverted	CHB HBeAg+ active disease	CC	DC	HCC	LT1	LT2	Dead+
**HBsAg seroconverted**	#	0	0	0	0	0.00005	0	0	0
**HBeAg seroconverted**	0.02	#	0.03	0.01	0	0.005	0	0	0
**CHB HBeAg+ active disease no treatment**	0.0175	0.05	#	0.05	0	0.005	0	0	0.0035
**CHB HBeAg+ active disease or CC on treatment**									
**Treatment response with peglyated interferon**	0.0175	0.20	#	0.05	0	0.005	0	0	0.0035
**Treatment response with tenofovir**	0.0175	0.054	#	0.05	0	0.005	0	0	0.0035
**Compensated cirrhosis (CC) no treatment**	0	0.05	0	#	0.05	0.025	0	0	0.051
**Decompensated cirrhosis (DC)**	0	0	0	0	#	0.025	0.03	0	0.39
**Hepatocellular carcinoma (HCC)**	0	0	0	0	0	#	0	0	0.56
**Liver transplant – first year (LT1)**	0	0	0	0	0	0	#	0	0.21
**Liver transplant – subsequent years (LT2)**	0	0	0	0	0	0	0	#	0.057

+an age-adjusted general population mortality is added to this amount; #, indicates the residual row probability; all values are converted to a Dirichlet distribution by assuming an effective sample size of 200 for each row(3)

For peer review only

Supplementary Table 2: Annual transition probability matrix for people who enter the model as HBsAg+ and HBeAg- derived from Shepherd(1) and Marcellin(2)

To: From:	HBsAg seroconverted	HBeAg seroconverted	CHB HBeAg- active disease	CC	DC	HCC	LT1	LT2	Dead+
**HBsAg seroconverted**	#	0	0	0	0	0.00005	0	0	0
**HBeAg seroconverted**	0.0175	#	0.03	0.01	0	0.005	0	0	0
**CHB HBeA- active disease no treatment**	0	0.015	#	0.05	0	0.005	0	0	0.0035
**CHB HBeAg- active disease or CC on treatment**									
**Treatment response with peglyated interferon**	0	0.75	#	0.05	0	0.005	0	0	0.0035
**Treatment response with tenofovir**	0	0.023	#	0.05	0	0.005	0	0	0.0035
**Decompensated cirrhosis (DC)**	0	0	0	0	#	0.025	0.03	0	0.39
**Hepatocellular carcinoma (HCC)**	0	0	0	0	0	#	0	0	0.56
**Liver transplant – first year (LT1)**	0	0	0	0	0	0	#	0	0.21
**Liver transplant – subsequent years (LT2)**	0	0	0	0	0	0	0	#	0.057

+an age-adjusted general population mortality is added to this amount; #, indicates the residual row probability; all values are converted to a Dirichlet distribution by assuming an effective sample size of 200 for each row(3)

For peer review only

Supplementary Table 3: Utility values from Shepherd(1) and Takeda(4)

Utility	Mean decrement	95% interval of sampled range^
**Age**		-
**0-44**	0.09	-
**45-54**	0.15	-
**55-64**	0.20	-
**65-74**	0.22	-
**75+**	0.27	-
**HBsAg-**	0	-
**HBeAg+ seroconverted / HBeAg- ALT/DNA low**	0	-
**HBeAg+ / HBeAg- active disease**	0.04	0.023-0.062
**Compensated cirrhosis**	0.44	0.34-0.55
**Decompensated cirrhosis**	0.54	0.43-0.73
**Hepatocellular carcinoma**	0.54	0.43-0.73
**Liver transplant (first year)**	0.54	0.43-0.73
**Liver transplant (subsequent years)**	0.32	0.22-0.43

Utility values are calculated by subtracting appropriate decrements from 1; ^Sampled values from the probabilistic sensitivity analysis using a beta distribution

For peer review only

Supplementary Figure 1. Cost-effectiveness acceptability curve for the base-case 2% HBsAg prevalence.

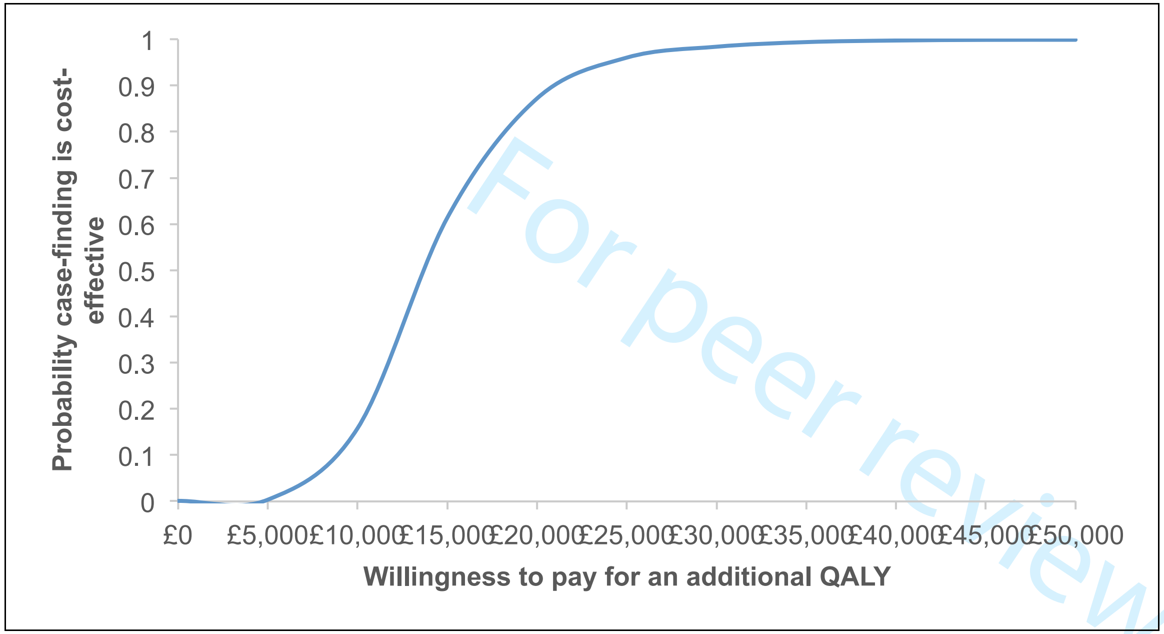

For peer review only

Reference

1. Shepherd J, Jones J, Takeda A, Davidson P, Price A. Adefovir dipivoxil and pegylated interferon alfa-2a for the treatment of chronic hepatitis B: a systematic review and economic evaluation. Health Technology Assessment. 2006;10(28).
2. Marcellin P, Gane E, Buti M, Afdhal N, Sievert W, Jacobson IM, et al. Regression of cirrhosis during treatment with tenofovir disoproxil fumarate for chronic hepatitis B: a 5-year open-label follow-up study. Lancet. 2013 Feb 9;381(9865):468-75. PubMed PMID: 23234725. Epub 2012/12/14. eng.
3. Briggs A, Sculpher M, Claxton K. Decision modelling for health economic evaluation. Gray A, Briggs A, editors. Oxford: Oxford University Press; 2006.
4. Takeda A, Jones J, Shepherd J, Davidson P, Price A. A systematic review and economic evaluation of adefovir dipivoxil and pegylated interferon-alpha-2a for the treatment of chronic hepatitis B. Journal of Viral Hepatitis. 2007;14:75-88.

For peer review only

CHEERS Checklist
Items to include when reporting economic evaluations of health interventions

The **ISPOR CHEERS Task Force Report**, *Consolidated Health Economic Evaluation Reporting Standards (CHEERS)—Explanation and Elaboration: A Report of the ISPOR Health Economic Evaluations Publication Guidelines Good Reporting Practices Task Force*, provides examples and further discussion of the 24-item CHEERS Checklist and the CHEERS Statement. It may be accessed via the *Value in Health* or via the ISPOR Health Economic Evaluation Publication Guidelines – CHEERS: Good Reporting Practices webpage: http://www.ispor.org/TaskForces/EconomicPubGuidelines.asp

Section/item	Item No	Recommendation	Reported on page No/ line No
**Title and abstract**			
Title	1	Identify the study as an economic evaluation or use more specific terms such as "cost-effectiveness analysis", and describe the interventions compared.	Pg 1/Line 1-2
Abstract	2	Provide a structured summary of objectives, perspective, setting, methods (including study design and inputs), results (including base case and uncertainty analyses), and conclusions.	Pg 3/Line 1-25
**Introduction**			
Background and objectives	3	Provide an explicit statement of the broader context for the study.<br>Present the study question and its relevance for health policy or practice decisions.	Pg 6/Line 5-10
**Methods**			
Target population and subgroups	4	Describe characteristics of the base case population and subgroups analysed, including why they were chosen.	Pg 8/lines 4-6
Setting and location	5	State relevant aspects of the system(s) in which the decision(s) need(s) to be made.	Pg 8/lines 4-6
Study perspective	6	Describe the perspective of the study and relate this to the costs being evaluated.	Pg 7/line 16
Comparators	7	Describe the interventions or strategies being compared and state why they were chosen.	Pg 8/lines4-15
Time horizon	8	State the time horizon(s) over which costs and consequences are being evaluated and say why appropriate.	Pg 7/line 17
Discount rate	9	Report the choice of discount rate(s) used for costs and outcomes and say why appropriate.	Pg 7/line 19
Choice of health outcomes	10	Describe what outcomes were used as the measure(s) of benefit in the evaluation and their relevance for the type of analysis performed.	pg 7/lines 18-19
Measurement of effectiveness	11a	*Single study-based estimates:* Describe fully the design features of the single effectiveness study and why the single study was a sufficient source of clinical effectiveness data.	pg 13, lines 8-12

For peer review only

	11b	*Synthesis-based estimates:* Describe fully the methods used for identification of included studies and synthesis of clinical effectiveness data.	n/a
Measurement and valuation of preference based outcomes	12	If applicable, describe the population and methods used to elicit preferences for outcomes.	pg 14, lines 1-3
Estimating resources and costs	13a	*Single study-based economic evaluation:* Describe approaches used to estimate resource use associated with the alternative interventions. Describe primary or secondary research methods for valuing each resource item in terms of its unit cost. Describe any adjustments made to approximate to opportunity costs.	pg 14, lines 7-13
	13b	*Model-based economic evaluation:* Describe approaches and data sources used to estimate resource use associated with model health states. Describe primary or secondary research methods for valuing each resource item in terms of its unit cost. Describe any adjustments made to approximate to opportunity costs.	pg 14, lines 3-7
Currency, price date, and conversion	14	Report the dates of the estimated resource quantities and unit costs. Describe methods for adjusting estimated unit costs to the year of reported costs if necessary. Describe methods for converting costs into a common currency base and the exchange rate.	pg 14, lines 5-7
Choice of model	15	Describe and give reasons for the specific type of decision-analytical model used. Providing a figure to show model structure is strongly recommended.	pg 8, lines 18-19
Assumptions	16	Describe all structural or other assumptions underpinning the decision-analytical model.	pg 8-10
Analytical methods	17	Describe all analytical methods supporting the evaluation. This could include methods for dealing with skewed, missing, or censored data; extrapolation methods; methods for pooling data; approaches to validate or make adjustments (such as half cycle corrections) to a model; and methods for handling population heterogeneity and uncertainty.	pg 14 lines 15-24, pg 15 lines 1-2
**Results**			
Study parameters	18	Report the values, ranges, references, and, if used, probability distributions for all parameters. Report reasons or sources for distributions used to represent uncertainty where appropriate. Providing a table to show the input values is strongly recommended.	pg 21
Incremental costs and outcomes	19	For each intervention, report mean values for the main categories of estimated costs and outcomes of interest, as well as mean differences between the comparator groups. If applicable, report incremental cost-effectiveness ratios.	pg 15, lines 5-10
Characterising uncertainty	20a	*Single study-based economic evaluation:* Describe the effects of sampling uncertainty for the estimated incremental cost and incremental effectiveness parameters, together with the impact	pg 15, line 11-24, pg 16 lines 1-11

For peer review only

		of methodological assumptions (such as discount rate, study perspective).	
	20b	*Model-based economic evaluation:* Describe the effects on the results of uncertainty for all input parameters, and uncertainty related to the structure of the model and assumptions.	n/a
Characterising heterogeneity	21	If applicable, report differences in costs, outcomes, or cost-effectiveness that can be explained by variations between subgroups of patients with different baseline characteristics or other observed variability in effects that are not reducible by more information.	pg 15, lines 21-24, pg 16, lines 1-2
**Discussion**			
Study findings, limitations, generalisability, and current knowledge	22	Summarise key study findings and describe how they support the conclusions reached. Discuss limitations and the generalisability of the findings and how the findings fit with current knowledge.	pg 16, lines 13-19
**Other**			
Source of funding	23	Describe how the study was funded and the role of the funder in the identification, design, conduct, and reporting of the analysis. Describe other non-monetary sources of support.	pg 2, lines 2-13
Conflicts of interest	24	Describe any potential for conflict of interest of study contributors in accordance with journal policy. In the absence of a journal policy, we recommend authors comply with International Committee of Medical Journal Editors recommendations.	

For consistency, the CHEERS Statement checklist format is based on the format of the CONSORT statement checklist

The **ISPOR CHEERS Task Force Report** provides examples and further discussion of the 24-item CHEERS Checklist and the CHEERS Statement. It may be accessed via the *Value in Health* link or via the ISPOR Health Economic Evaluation Publication Guidelines – CHEERS: Good Reporting Practices webpage: http://www.ispor.org/TaskForces/EconomicPubGuidelines.asp

The citation for the CHEERS Task Force Report is:
Husereau D, Drummond M, Petrou S, et al. Consolidated health economic evaluation reporting standards (CHEERS)—Explanation and elaboration: A report of the ISPOR health economic evaluations publication guidelines good reporting practices task force. Value Health 2013;16:231-50.

For peer review only
